# Molecular Diversity of Linear Peptides Revealed by Transcriptomic Analysis of the Venom Gland of the Spider *Lycosa poonaensis*

**DOI:** 10.3390/toxins14120854

**Published:** 2022-12-03

**Authors:** Alhussin Mohamed Abdelhakeem Megaly, Masahiro Miyashita, Mohammed Abdel-Wahab, Yoshiaki Nakagawa, Hisashi Miyagawa

**Affiliations:** 1 Division of Applied Life Sciences, Graduate School of Agriculture, Kyoto University, Kyoto 606-8502, Japan; 2 Zoology Department, Faculty of Science, Al-Azhar University, Assuit 71524, Egypt

**Keywords:** spider, linear peptide, transcriptomics, antibacterial activity, insect toxicity, α-helical structure

## Abstract

Spider venom is a complex mixture of bioactive components. Previously, we identified two linear peptides in *Lycosa poonaensis* venom using mass spectrometric analysis and predicted the presence of more linear peptides therein. In this study, a transcriptomic analysis of the *L. poonaensis* venom gland was conducted to identify other undetermined linear peptides in the venom. The results identified 87 contigs encoding peptides and proteins in the venom that were similar to those in other spider venoms. The number of contigs identified as neurotoxins was the highest, and 15 contigs encoding 17 linear peptide sequences were identified. Seven peptides that were representative of each family were chemically synthesized, and their biological activities were evaluated. All peptides showed significant antibacterial activity against Gram-positive and Gram-negative bacteria, although their selectivity for bacterial species differed. All peptides also exhibited paralytic activity against crickets, but none showed hemolytic activity. The secondary structure analysis based on the circular dichroism spectroscopy showed that all these peptides adopt an amphiphilic α-helical structure. Their activities appear to depend on the net charge, the arrangement of basic and acidic residues, and the hydrophobicity of the peptides.

## 1. Introduction

Spiders (Araneae), which first evolved around 300 million years ago, are one of the most successful groups of organisms on Earth [1]. According to the World Spider Catalog, more than 50,000 species are found worldwide (https://wsc.nmbe.ch/, accessed on 28 October 2022). The evolutionary success of spiders can be attributed partly to their silk web, which was developed to capture or wrap prey [2]. In addition to webs, spiders use venom to subdue prey (insects) and to defend themselves against predators (mammals) [3]. Spider venom is a complex mixture of bioactive components, and interest in spider venom as a potential source of novel agrochemicals and pharmaceuticals is growing [4,5].

Spider venom components are typically divided into four groups according to their chemical nature: (1) low molecular mass compounds, (2) linear peptides without disulfide bridges, (3) cysteine-rich peptides, and (4) proteins and enzymes [3]. Among these, peptides play an essential role in prey capture and predator defence, performing various functions, including neurotoxic, analgesic, cytotoxic, and necrotic activities [6]. Spider venoms typically contain various cysteine-rich peptides with conserved structural motifs, such as an inhibitor cystine knot (ICK) motif [3,7]; most cysteine-rich peptides have evolved as a neurotoxin to effectively act on specific ion channels or receptors in target organisms. Linear peptides without disulfide bridges are also known to perform critical functions in venom, although they have only been identified from a few spider families [8]. Many linear peptides have been identified as antimicrobial peptides (AMPs), which may prevent pathogen infection during the injection of venom into prey or ingestion of prey [9,10]. Their cationic and amphipathic α-helical structure plays a vital role in interaction with the anionic surface of bacterial membranes to disrupt the integrity of the cellular membrane [11]. In addition, some linear peptides not only exhibit antimicrobial activity but also contribute directly or synergistically to the insecticidal activity of the venom [12].

Recently, a comprehensive analysis of linear peptides was conducted to determine their existence in the venom of various spider species [8]. The venom glands of spiders of 23 families were analyzed via transcriptomic analysis, and linear peptides were found in spiders of five families and the genus *Cupiennius* among them. In particular, many linear peptides have been identified in spiders of the Lycosidae family, suggesting that linear peptides hold some critical functions in the venom of these spiders. We recently identified two linear peptides in *Lycosa poonaensis* venom using mass spectrometric analysis [13]. According to recent findings, there are likely other undetermined linear peptides in the venom of that spider [8]. In this study, *de novo* transcriptomic analysis of the venom gland of *L. poonaensis* was performed to estimate the structures of all peptides and proteins expressed in the venom. The analysis revealed 17 linear peptides in total, and seven representative peptides were chemically synthesized in order to evaluate their biological activities.

## 2. Results and Discussion

### 2.1. Transcriptomic Analysis of the Venom Gland of L. poonaensis

**Figure 1:**
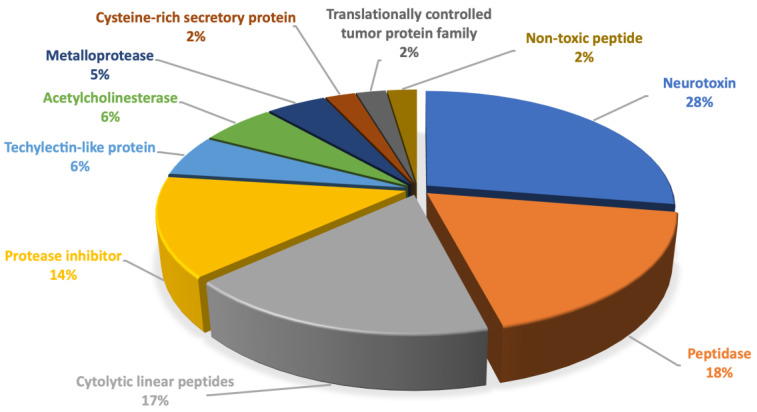
The proportions of the components identified by transcriptomic analysis of the venom gland of *L. poonaensis*.

A total of 33,055,014 raw reads were obtained through sequencing. After adapter- and quality-trimming, the clean reads (32,712,326) were assembled in a de novo fashion using Trinity; this resulted in 280,691 contigs with N50 of 743 bp (186–34,812 bp). A BLASTP search was performed for coding regions predicted from the contigs against a database of reported sequences identified from the spider venom. A total of 87 contigs encoding similar peptides and proteins to those of other spider venoms were identified, as shown in Table S1. The number of sequences identified as neurotoxins was the highest, followed by peptidases, cytolytic linear peptides, and other enzymes or proteins (Figure 1).

#### 2.1.1. Peptides Other Than Linear Cytolytic Peptides

**Figure 2:**
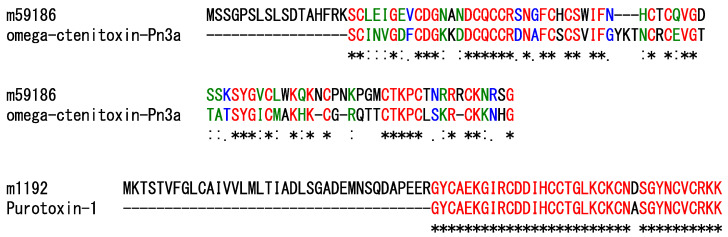
Comparison of the sequences of peptide precursors similar to neurotoxins. Asterisks (*), colons (:), and periods (.) indicate identical (shown in red), strongly similar (green), and weakly similar residues (blue), respectively.

Neurotoxins are the main active components of this spider’s venom [14,15]. In the current study, 24 contigs contain ORFs encoding peptide precursors that share sequence similarities to peptides with possible neurotoxic effects; among these, two contigs encode sequences similar to the spider venom peptides that were experimentally confirmed to act on ion channels. The contig m.59186 encodes a precursor similar to omega-ctenitoxin-Pn3a, which was identified in *Phoneutria nigriventer* (Figure 2). Omega-ctenitoxin-Pn3a is known to irreversibly block calcium currents generated by Cav2.1 and Cav2.2 channels, although it showed a partial and reversible inhibitory effect on that generated by the Cav2.3 channel [16]. The contig m.1192 encodes a precursor similar to purotoxin-1, identified in *Alopecosa marikovskyi* (Figure 2). Purotoxin-1 displays a potent and selective inhibitory action on the P2 × 3 receptors expressed in mammalian sensory neurons [17,18]. Other contigs also encode precursors similar to neurotoxins, but their activities have not yet been experimentally evaluated.

**Figure 3:**
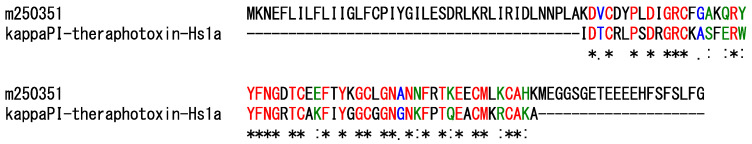
Comparison of the sequences of peptides similar to protease inhibitors. Asterisks (*), colons (:), and periods (.) indicate identical (shown in red), strongly similar (green), and weakly similar residues (blue), respectively.

Protease inhibitor peptides are often identified in spider venom [19], and it has been proposed that these peptides could protect the integrity of other venom components through their inhibitory activity on proteases. This study found that four contigs contain ORFs encoding precursors similar to Kunitz-type protease inhibitor peptides [20], and two contigs contain those similar to a cysteine proteinase inhibitor and a Kazal-type serine protease inhibitor [21]. Of these, the contig m.250351 contains an ORF encoding a precursor similar to kappaPI-theraphotoxin-Hs1a, identified in *Cyriopagopus schmidti* (Figure 3). Interestingly, this toxin performs a dual function as a trypsin inhibitor and a potassium channel blocker [19].

**Figure 4:**
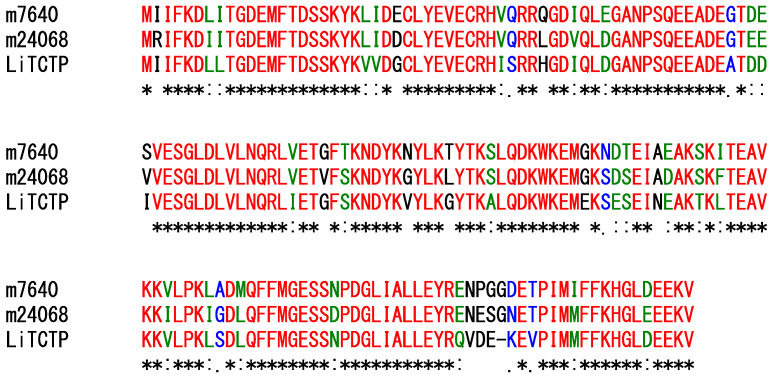
Comparison of the sequences of proteins similar to TCTPs. Asterisks (*), colons (:), and periods (.) indicate identical (shown in red), strongly similar (green), and weakly similar residues (blue), respectively.

Translationally controlled tumor protein homologs (TCTPs) are known to be a histamine-releasing factor, promoting allergic responses in mammalian tissues by inducing the release of histamine from basophils or mast cells [22]. TCTPs have been observed in the venoms of some spider species, but their biological role in prey capture remains unknown. In this study, two contigs (m.7640 and m.24068) encode precursors similar to TCTPs (Figure 4).

Furthermore, eight contigs contain ORFs encoding precursors similar to toxin-like peptides, the functions of which are not yet understood.

#### 2.1.2. Enzymes and Proteins

Spider venom also contains enzymes and proteins, which can be classified into three functional classes: (1) enzymes acting as a spreading factor; (2) proteins with a function in the venom gland, including the maturing of toxins; and (3) proteins directly acting on targets of the prey organism [7]. However, the biological roles of venom proteins have not been experimentally confirmed in most cases, and their functions have been predicted based on their similarity to other known proteins. In the current study, 32 contigs contain ORFs encoding precursors enzymes or proteins, which were previously reported to be expressed in the venom gland.

The enzymes acting as a spreading factor can degrade or modify the extracellular matrix or the cell membrane, which can facilitate the movement of toxins in the prey organism; the main group of enzymes responsible for this function is matrix-metalloproteases [23]. Four contigs contain ORFs encoding precursors similar to the Astacin-like metalloprotease identified in *Loxosceles intermedia* [24].

Peptidases play a critical role in venom, specifically in producing mature peptides, by cleaving the precursor through recognition of the specific motif [25]. In this study, 16 contigs contain ORFs encoding precursors similar to peptidases; some of them may be involved in the maturation of linear peptides, as discussed in the following section.

Acetylcholinesterases are known to exist in some spider venoms, possibly acting on synaptic targets of the prey organism [21]. In this study, five contigs contain ORFs encoding precursors similar to acetylcholinesterases identified in spider venom. 

Tachylectin-like proteins are hypothesized to be involved in the protection of the venom gland against microbial infections [26]; five contigs in this study encode precursors similar to tachylectin-like proteins from spider venom. 

Cysteine-rich secretory proteins (CRISPs) are widespread in various animal venoms, although their functions remain unclear [27]. In this study, two contigs encode precursors similar to CRISPs.

#### 2.1.3. Cytolytic Linear Peptides

Cytolytic linear peptides play an important role in the venom of several spider families [8]. It was reported that linear peptides were found in spiders in the RTA clade. In this study, 15 contigs were found to contain ORFs encoding precursors similar to cytolytic linear peptides. Sequences similar to LyeTx I were observed in six precursors, and those similar to M-lycotoxin-Hc2a were in four precursors. LyeTx I, identified in *Lycosa erythrognatha*, shows antimicrobial and hemolytic activities [28]; M-lycotoxin-Hc2a, identified in *Hogna carolinensis*, is known to perform various biological activities, including antimicrobial, cytolytic, hemolytic, and neurotoxic actions [29,30]. The sequence of lyp2370, which was identified in a previous study [13], was found in a precursor encoded in the contig m.2773, while the sequence of lyp1987 (the same sequence as M-lycotoxin-Ls3b identified in *Lycosa singoriensis*) was found in precursors encoded in six contigs. In addition, the sequences of lyp2370 and lyp1987 are repeatedly present in a single precursor encoded in contigs m.2773 and m.2678. This type of precursor structure is observed in linear peptides in other spider venoms [31]. In general, precursors of cytolytic linear peptides in spider venom are composed of a short signal sequence followed by propeptide and mature regions. The propeptide and mature regions are separated by specific motifs, known as a processing quadruplet motif (PQM) and an inverted processing quadruplet motif (iPQM). Peptidases recognize these motifs in a precursor sequence to produce mature peptides [32]: the N-terminal side of mature regions is cleaved by recognition of the PQM, and their C-terminal side is cleaved based on the iPQM. The PQM contains an Arg residue at position -1 and Glu residues at positions -2, -3, and/or -4 (XXXR, where any of X = E); the iPQM is a mirrored PQM consisting of an Arg residue at position 1 and Glu residues at positions 2, 3, and/or 4 (RXXX, where any of X = E) [33]. In addition to a simple precursor that contains one mature region, one precursor sequence of linear peptides in spider venom can have two or more mature regions: these are known as binary or complex precursors [31].

**Figure 5:**
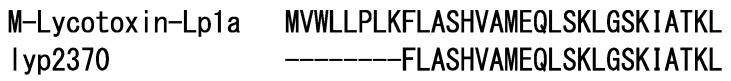
Comparison of the sequences between M-Lycotoxin-Lp1a and lyp2370.

In this study, 17 possible cytolytic linear peptides were identified from precursors encoded in 15 contigs based on the PQM and iPQM cleavage sites (Table 1 and Table 2). Of these simple, binary, and complex types of precursors, four types of PQM (EEAR, EEER, ELER, and GEER) and nine types of iPQM (RSED, REDS, REEG, REEI, REEN, REES, RNEE, RNEQ, and RSEE) were observed. The lengths of the linker sequences range from 6–18 amino acid residues. Most of the mature sequences contain Gly residues at the C-terminus, which is likely to be amidated. These cytolytic linear peptides can be divided into six families according to sequence similarity (Table 2). The sequence of lyp1987 identified in our previous study was identical to that of M-lycotoxin-Lp2 [13]. On the other hand, the sequence of lyp2370 was shared with that of M-lycotoxin-Lp1a but lacked its N-terminal region (Figure 5); this may be due to the additional cleavage of the N-terminal region of M-lycotoxin-Lp1a in the venom. Here, we renamed lyp1987 and lyp2370 to M-Lycotoxin-Lp2 and M-lycotoxin-Lp1a(9-30), respectively.

In order to confirm the presence of these identified peptides in the venom, a high-resolution LC/MS analysis was performed. This analysis revealed that ten peptides were detected in the venom (Table 2 and Figure S1). Interestingly, only short versions of linear peptides, in which the N-terminal region was truncated as observed for M-lycotoxin-Lp1a, were found for M-lycotoxin-Lp1b, M-lycotoxin-Lp3c, and M-lycotoxin-Lp3d (Table 2 and Figure S1). It is possible that additional enzymatic cleavage occurs in the venom, as observed in scorpion venom [34], although degradation by peptidases derived from tissues other than the venom gland during the venom extraction cannot be ruled out. Regarding the peptides that were not detected in this study, it is possible that their expression levels are extremely low or that further cleavage might have occurred. It could also be due to errors in the de novo assembly.

**Figure 6:**
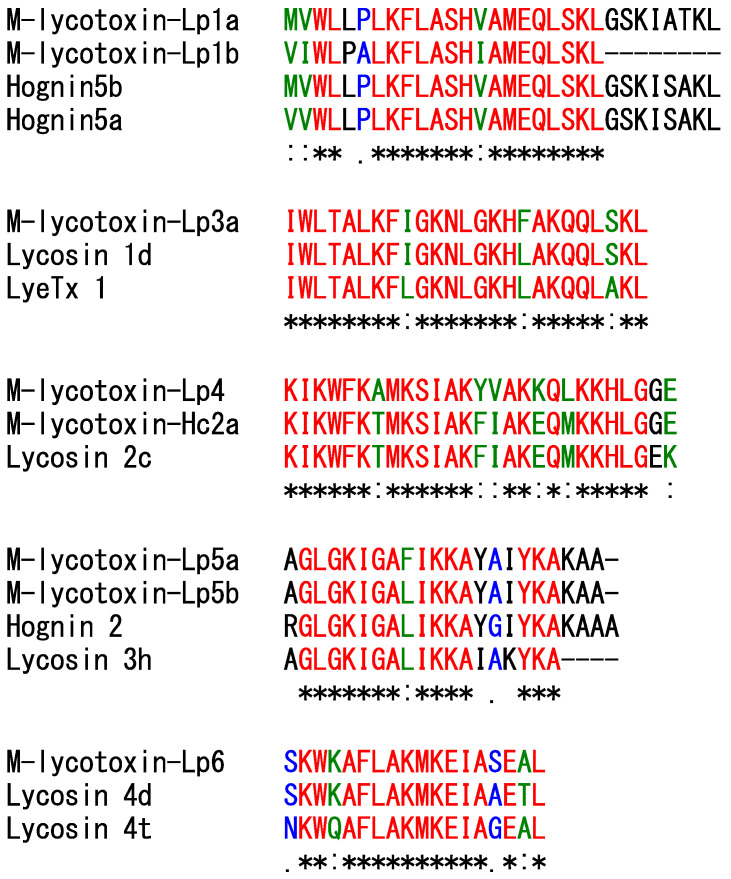
Multiple-sequence alignment of the synthesized peptides with their similar peptides. Asterisks (*), colons (:), and periods (.) indicate identical (shown in red), strongly similar (green), and weakly similar residues (blue), respectively.

Seven peptides, which were detected in the venom by LC/MS analysis, were selected for biological characterization (Table 3). Although only a short version of M-lycotoxin-Lp1b was detected in the venom, the activity of its full-length version was evaluated in this study. This is because M-lycotoxin-Lp1a(9-30), a shorter version of M-lycotoxin-Lp1a, showed low activity. As shown in Figure 6, M-lycotoxin-Lp3a and M-lycotoxin-Lp4 show sequence similarities to LyeTx I and M-lycotoxin-Hc2a, respectively, whose activity has already been reported [28,29,30]. At the same time, M-lycotoxin-Lp1a, M-lycotoxin-Lp5a, and M-lycotoxin-Lp6 show similarities to hognin 5b, hognin 2, and lycosin 4d, respectively, whose activity has not yet been reported. In addition, M-lycotoxin-Lp1b has a sequence lacking the C-terminal region of M-lycotoxin-Lp1a. M-lycotoxin-Lp5b differs from M-lycotoxin-Lp5a only at the ninth residue. In a comparison of the activities of these peptides, the effect of sequence differences on activity will be elucidated.

**Figure 7:**
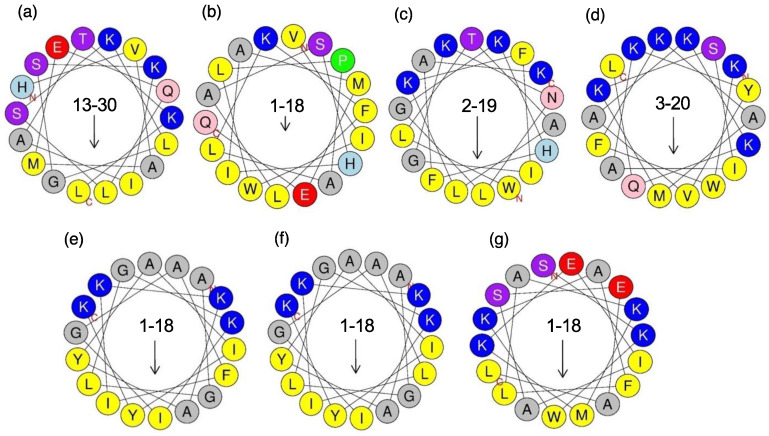
Helical wheel projections. (**a**) M-lycotoxin-Lp1a, (**b**) M-lycotoxin-Lp1b, (**c**) M-lycotoxin-Lp3a, (**d**) M-lycotoxin-Lp4, (**e**) M-lycotoxin-Lp5a, (**f**) M-lycotoxin-Lp5b, and (**g**) M-lycotoxin-Lp6. Arrows indicate hydrophobic moments. Hydrophobic residues are shown in yellow, serine and threonine in purple, basic residues in blue, acidic residues in red, asparagine and glutamine in pink, alanine and glycine in grey, histidine in light blue, and proline in green. N and C shown in red denote the N- and C-termini in this region, respectively.

All synthesized peptides were analyzed using CD spectroscopy to estimate their secondary structures in solution (Figure S2). The results demonstrate that all these peptides are assumed to adopt an α-helical structure in 50% trifluoroethanol, a helix-promoting solvent (Table 4). In addition, these peptides are likely to adopt an amphipathic nature based on the helical wheel projection (Figure 7).

### 2.2. Biological Activities

The antibacterial activity of the synthesized peptides was evaluated using Gram-negative (*E. coli*) and Gram-positive (*S. aureus* and *B. subtilis*) bacteria. In general, linear peptides that adopt an amphipathic α-helical structure are known to show antimicrobial activity by electrically attracting to negatively charged groups of the bacterial membranes, resulting in the formation of transient pores, membrane perturbation, and cell lysis [11]. Comparison of the activity of these peptides may provide structural factors important for their antibacterial activity against the three bacterial species. As shown in Table 5, all peptides showed significant antibacterial activity against the three bacteria (except for M-lycotoxin-Lp6, which showed no activity against *S. aureus* even at 100 µM). Of these, M-lycotoxin-Lp4 showed the highest activity against *E. coli*: this peptide has the highest net charge (+8) among the peptides tested in this study, which may account for its high activity [36,37]. At the same time, M-lycotoxin-Lp3a showed the highest activity against *S. aureus* and *B. subtilis*: M-lycotoxin-Lp3a has a relatively high net charge (+6) and a relatively high hydrophobicity, both of which may contribute to the relatively high activity of M-lycotoxin-Lp3 against *S. aureus* and *B. subtilis*. A similar activity profile is observed between M-lycotoxin-Lp1a and M-lycotoxin-Lp1b, in which the former is more active against *E. coli* and the latter against *S. aureus* and *B. subtilis*. A moderately high net charge (+4) of M-lycotoxin-Lp1a may contribute to its relatively high activity against *E. coli*; in contrast, M-lycotoxin-Lp1b has the highest hydrophobicity and a low net charge (+2) due to its lack of the C-terminal region seen in M-lycotoxin-Lp1a. These results suggest that the balance between net charge and hydrophobicity may affect the selectivity against these bacteria, although a complete understanding of the relationship between the structure and antibacterial activity of AMPs requires validation with synthetic analogs [38].

**Figure 8:**
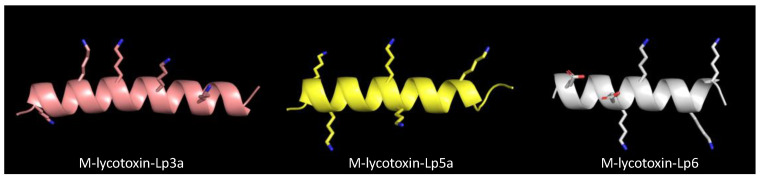
Comparison of the arrangement of basic (Lys) and acidic (Glu) residues on the hydrophilic surfaces of M-lycotoxin-Lp3a, M-lycotoxin-Lp5a, and M-lycotoxin-Lp6. Hydrophilic surfaces are facing on the visible side.

M-lycotoxin-Lp5a, M-lycotoxin-Lp5b, and M-lycotoxin-Lp6 showed relatively high activity against *E. coli* and *B. subtilis* but very weak or no activity against *S. aureus*. These peptides have a high net charge (+3 or +6) and sufficient hydrophobicity to exhibit activity. Thus, other reasons may cause their low activity against *S. aureus*. The arrangement of basic residues (Lys) on the polar surface of their α-helical structure was compared with that of M-lycotoxin-Lp3a, based on their 3D structures predicted using AlphaFold2 [39] (Figure 8). Lys residues are clustered in the center of the polar surface of M-lycotoxin-Lp3a: in contrast, Lys residues are located near the boundary of the polar surfaces of M-lycotoxin-Lp5a, and M-lycotoxin-Lp6. This difference may affect the selectivity among bacteria species, although the detailed mechanism is unclear [40]. The extremely low activity of M-lycotoxin-Lp6 could be due to the presence of two acidic residues (Glu) on its polar surface (Figure 8). No apparent differences in activity were observed between M-lycotoxin-Lp5a and M-lycotoxin-Lp5b, indicating that the type of hydrophobic residue (Phe vs. Leu) at this position has a negligible effect.

We previously found that M-lycotoxin-Lp1a(9-30) has only weak antibacterial activity, although it adopts an amphipathic α-helical structure that is typical of antimicrobial peptides [13]. As mentioned above, M-lycotoxin-Lp1a(9-30) has a sequence lacking the N-terminal region (MVWLLPLK) seen in M-lycotoxin-Lp1a, which contains many hydrophobic residues. In addition, a Pro residue can increase antibacterial activity by introducing a kink in the α-helical structure [41]. Thus, the N-terminal region in M-lycotoxin-Lp1a is likely to contribute significantly to antibacterial activity.

In a subsequent step, the insect toxicity of these peptides was examined (Table 5). Cytolytic linear peptides are known to show toxicity against insects by disturbing cell membranes and dissipating ion and voltage gradients across nerve membranes [30]. All peptides exhibited paralytic activity against crickets; of the tested peptides, M-lycotoxin-Lp4 showed the highest toxicity (PD_50_ = 10 µg/g), while M-lycotoxin-Lp6 showed the lowest toxicity (PD_50_ = 94 µg/g). This result may reflect differences in the net charge between these peptides (+8 vs. +3). However, M-lycotoxin-Lp1b, which has the lowest net charge (+2), exhibited relatively high toxicity (PD_50_ = 31 µg/g). Since M-lycotoxin-Lp1b contains a Pro residue at the N-terminal region, its activity may be affected by a bent structure caused by the Pro residue, as discussed above [41]. M-Lycotoxin-Lp5a and M-lycotoxin-Lp5b showed similar insect toxicity, indicating that the difference in the ninth residue does not affect the activity. Linear peptides are known not only to exhibit insect toxicity but also to enhance the activity of other insecticidal neurotoxins [42]. The presence of such multifunctional linear peptides in spider venom may be advantageous for the survival of some spider species with a low economic cost [8]. Although the activity of neurotoxins in this spider venom has not yet been evaluated, it is worthwhile to examine the potentiating effect of the linear peptides in the future.

Finally, the hemolytic activity of these peptides was examined (Table 5); none of them showed activity, even at 100 µM. It has been reported that the hydrophobicity of peptides, rather than their net charge, is important to the expression of hemolytic activity [43] due to the more neutral and hydrophobic properties of the mammalian cell membranes, which are primarily composed of zwitterionic phospholipids and cholesterol. The peptides tested in this study may have relatively low hydrophobicity, so they cannot interact with erythrocyte membranes.

## 3. Conclusions

In this study, transcriptomic analysis was conducted of the *L. poonaensis* spider’s venom gland. In total, 87 contigs encoding peptides and proteins were identified, and these are assumed to perform biological functions in the venom. The highest number of contigs were annotated as neurotoxins, but 15 contigs containing 17 linear peptides were also identified. Seven representative peptides were chemically synthesized, and their biological activities were analyzed. All peptides showed significant antibacterial activity and insect toxicity, but none showed hemolytic activity; their activities likely depend on the net charge, the arrangement of basic/acidic residues, and the hydrophobicity of the amphiphilic α-helical structure of the peptides. However, since their structural variation is limited, further validation study is necessary using synthesized analogs with amino acid substitutions, which will be done in future studies. The information obtained in this study not only improves our understanding of the structural factors involved in linear peptides’ expression of antimicrobial activity but also provides important insight into the role of linear peptides in the venom of spiders of the family Lycosidae.

## 4. Material and Methods

### 4.1. Biological Materials

*L. poonaensis* spiders were collected from the Western Desert of Egypt. Venom glands were dissected from two specimens, which were anesthetized on ice, and directly placed in a microtube containing RNAlater (Thermo Fisher Scientific, Waltham, MA, USA) at room temperature for 1 h, followed by storage at −80 °C until RNA extraction.

### 4.2. RNA Extraction

Venom glands were manually homogenized in a glass tube of a microtissue grinder. The total RNA was extracted using the TRIzol reagent (Thermo Fisher Scientific) and further purified using the RNeasy Mini Kit (Qiagen, Venlo, The Netherlands) according to the manufacturer’s instructions. The concentration and purity of the total RNA samples were estimated using the Nanodrop Lite Spectrometer (Thermo Fisher Scientific).

### 4.3. Library Preparation and Sequencing

Library preparation and sequencing analysis were conducted at Bioengineering Lab. Co., Ltd. (Sagamihara, Japan). The concentration of the total RNA was measured using the Quantus Fluorometer with the QuantiFluor RNA system (Promega, Madison, WI, USA). The quality of the RNA was then analyzed using the 5200 Fragment Analyzer System (Agilent Technologies, Santa Clara, CA, USA). RNA sequencing libraries were prepared using the MGIEasy RNA Directional Library Prep Set (MGI Tech, Shenzhen, China) according to the manufacturer’s instructions. The concentration of the prepared library solution was determined using the Qubit 3.0 Fluorometer with the dsDNA HS Assay Kit (Thermo Fisher Scientific). The quality of the library was analyzed using the 5200 Fragment Analyzer System with the dsDNA 915 Reagent Kit (Agilent Technologies). Single-stranded circular DNA was prepared using the constructed library and the MGIEasy Circularization Kit (MGI Tech), and DNA nanoballs (DNBs) were prepared using the DNBSEQ-G400RS High-throughput Sequencing Kit (MGI Tech). The 200 bp paired-end sequencing was performed using the DNBSEQ-G400 (MGI Tech). The short-read data were deposited in the Read Archive of DDBJ (accession number DRA014441).

### 4.4. De Novo Assembly and Functional Annotations

After adapter trimming using Cutadapt (1.9.1), the reads were further trimmed using Sickle (1.33), with a minimum window quality score of 20; reads shorter than 40 bp after trimming were removed. The high-quality reads were assembled into contigs using Trinity (2.10.0). Coding regions were predicted with TransDecoder (5.5.0), and redundant sequences were removed by clustering using CD-HIT (4.8.1) at the threshold of 1.00. The predicted sequences were submitted to homology searches against a local BLAST database, which was constructed using sequences identified from spider venom (UniProt Animal Toxin Annotation Project) in order to annotate the functions of identified peptides and proteins. For each annotated sequence, a BLAST search was individually performed against the database within the class Arachnida (taxid: 6854). All annotated sequences were manually inspected so as to exclude internal and partial sequences. Annotated complete sequences were classified into groups based on their structure, and the percentage of the number of sequences in each group to the total number of sequences was calculated. The complete sequences were submitted to SignalP (5.0) to estimate the signal peptide sequence. Mature regions of linear peptides were predicted based on the presence of a processing quadruplet motif (PQM) and an inversed processing quadruplet motif (iPQM) [32]. Multiple sequence alignments were performed with CLUSTAL W [44].

### 4.5. Peptide Synthesis

Peptides were synthesized using the Fmoc-based solid-phase method with an automated microwave peptide synthesizer (Liberty Blue) (CEM, Matthews, NC, USA), in which *N*, *N’*-diisopropylcarbodiimide and Oxyma Pure were used for coupling reactions. Rink Amide ProTide Resin (LL) resin and Cl-MPA ProTide Resin (LL) resin (CEM) were used for the syntheses of C-terminal peptide amides and acids, respectively. The synthesized peptides were purified using reversed-phase HPLC (RP-HPLC) on a C18 column, and their identity was confirmed through LC/MS analyses, as shown below.

### 4.6. Mass Spectrometric Analysis

LC/MS measurements were carried out using an Orbitrap Exploris 240 mass spectrometer at 60,000 resolution (Thermo Fisher Scientific) for confirmation of peptides in the venom and an LCMS-2020 mass spectrometer (Shimadzu, Kyoto, Japan) for confirmation of peptide synthesis. For analysis by Orbitrap Exploris 240, an RP-HPLC column (Everest C18 1.0 mm × 250 mm, Hichrom, Reading, UK) was used for separation; this column was eluted with 0.1% formic acid in H_2_O (solvent A) and 0.1% formic acid in CH_3_CN (solvent B) at a flow rate of 0.05 mL/min using a linear gradient of 5–50% of solvent B over 45 min. For analysis by LCMS-2020, an RP-HPLC column (Poroshell 120-EC-C18 2.1 mm × 75 mm, Agilent) was used for separation; this column was eluted with 0.1% formic acid in H_2_O (solvent A), and 0.1% formic acid in CH_3_CN (solvent B) at a flow rate of 0.3 mL/min using a linear gradient of 5–60% of solvent B over 15 min.

### 4.7. Circular Dichroism (CD) Measurements

CD spectra were recorded on a J-820 spectropolarimeter (Jasco, Tokyo, Japan). The spectra were measured between 190–260 nm (in 0.2-nm steps) at 25 °C with a 1-mm-pathlength cell. Samples were dissolved in a 0.2 M phosphate buffer (pH 7.0) or 50% TFE in a 0.2 M phosphate buffer (pH 7.0) at a concentration of 10 µM. Five scans were averaged for each sample. The percentages of secondary structures were estimated from the spectra using the DichroWeb server [45]. The helical wheel projection was performed using the online software HeliQuest (https://heliquest.ipmc.cnrs.fr, accessed on 14 July 2022).

### 4.8. Antibacterial Activity

Antibacterial activity was measured using the Gram-negative bacteria *Escherichia coli* NBRC 3972 and the Gram-positive bacteria *Staphylococcus aureus* NBRC 13276 and *Bacillus subtilis* NBRC 3009 (NITE Biological Resource Center, Chiba, Japan), using a liquid growth inhibition assay. Each bacteria strain was grown in a liquid LB medium (1% tryptone, 0.5% yeast extract, and 1% NaCl). Aliquots of each sample (10 µL) were incubated with 90 µL of a suspension of a mid-logarithmic phase culture of bacteria in a 96-well plate at a starting absorbance of A_595_ = 0.001 in the LB medium; the incubation occurred overnight at 37 °C with continuous shaking. Growth inhibition was monitored by measuring the absorbance at 595 nm using a Benchmark microplate reader (Bio-Rad, Hercules, CA, USA). Experiments were repeated at least three times, each time in duplicate. Minimal inhibitory concentrations (MICs) are expressed as the interval of concentrations [a]–[b], where [a] is the highest concentration at which bacteria still grow and [b] is the lowest concentration causing growth inhibition.

### 4.9. Insect Toxicity

Insect toxicity was tested on crickets (*Acheta domesticus*, 50 ± 5 mg body weight) by injecting 1–2 µL of each sample dissolved in distilled water into their abdominal region. Distilled water was injected as a control. For each measurement, 10 insects were used, and the number of paralyzed insects was counted 30 min after injection. The doses required to induce paralysis in half of the test animals (PD_50_) were determined using the statistical software PRISM (GraphPad Software, La Jolla, CA, USA).

### 4.10. Hemolytic Activity

Fresh sheep red blood cells (sRBCs) were washed three times with PBS (35 mM phosphate buffer and 150 mM NaCl, pH 7.2) through centrifugation at 2000× *g* for 5 min and resuspended in PBS. Specifically, aliquots of each sample in PBS (50 μL) were added to 50 μL of sRBC suspension [4% (*v*/*v*) in the final] in a microtube and incubated for 1 h at 37 °C; then, the samples were centrifuged at 2000× *g* for 5 min. The supernatant was transferred to a 96-well plate to monitor hemoglobin release, which was done by measuring the absorbance of the supernatant at 450 nm using a Benchmark microplate reader. PBS and 0.1% Triton X-100 were used as the negative and positive controls, respectively. The experiments were repeated 3 times, each time in triplicate. In the equation to calculate the hemolysis rate, A_P_ refers to peptide sample absorbance, A_T_ refers to 0.1% Triton X-100 positive control absorbance, and A_0_ refers to PBS sample negative control absorbance.

$$\mathrm{Hemolysis}\;\mathrm{rate}\;(\%)=\frac{A_{p}-A_{0}}{A_{T}-A_{0}}\times 100$$

### 4.11. 3D Structure Prediction

3D structures of peptides were modeled with AlphaFold2 [39] using the ColabFold server [46].

## Figures and Tables

**Table 1:** Identification of mature peptides from the precursor sequences of linear peptides.

Precursor Type	Contig	Structure of Precursor
Simple	m.2649	<EEARIWLTALKFIGKNLGKHFAKQQLSKL **G** RSED >
	m.2799	<EEARIWLTALKFLGKNLGKHFAKQQLAKL **G** RSED >
Binary	m.2698	<EEARIWLTALKFIGKNLGKHFAKQQLSKL **G** RSED MTVNDD EEAR IWLTALKFIGKNLGKHFAKQQLSKL **G** RSED >
	m.2695	<EEARIWLTALKFIGKNLGKHFAKQQLSKL **G** RSED MTVNDD EEAR IWLTALKFLGKNLGLKFIGKNLGKHFAKQQLSKL **G** RSED >
	m.2781	<EEARIWLTALKFLGKNLGKHFAKQQLAKL **G** RSED ISENLSADDD EEAR IWLTALKFIGKNLLVNSIKIFR *
	m.2690	<EEARIWLTALKFIGKNLGKHFAKQQLSKL **G** RSED MTVNDD EEAR IWLTALKFLGKNLGLKFIGKNLGKH *
Complex	m.2667	<EEARGRLQAFLAKMKEIAAQTL **G** REEN LFAN EEER VIWLPALKFLASHIAMEQLSKL **G** RNEQ TP EEAR IWLTALKFIGKNLGKHFAKQQLSKL **G** RNEQ TP EEAR IWLTALKQLSKLGRT *
	m.2650	<EEARGRLQAFLAKMKEIAAQTL **G** REEN LFAN EEER VIWLPALKFLASHIAMEQLSKL **G** RNEQ TP EEAR IWLTALNKFIGKNLGKHFAKQQLSKL **G** RNEQ TP EEAR IWLTALKQLSKLGRT *
	m.2660	<EEARGRLQAFLAKMKEIAAQTL **G** REEN LFAN EEER VIWLPALKFLASHIAMEQLSKL **G** RNEQ TP EEAR IWLTALNKFL **G** *
	m.2706	<EEARGRLQAFLAKMKEIAAQTL **G** REEN LFAN EEER VIWLPALKFLASHIAMEQLSKL **G** RNEQ TP EEAR IWLTALNNASEAL **G** REEI >
	m.2678	<EEARSKWKAFLAKMKEIASEAL **G** REEG VSANED EEAR GRLQAFLAKMKEIAAQTL **G** REDS LSANED EEAR GRLQAFLAKMKEIAAQTL **G** REES LSENEN EEAR GRLQAFLAKMKEIAAQTL **G** REES VSANED EEAR SKWKAFLAKMKEIASEAL **G** REEN >
	m.2715	<EEARIWLTALKFIGKNLARIWLTALKFLGKNLGLKFIGKNLGKHFAKQQLSKL **G** RS EEAR IWLTALKFIGKNLGKHLAKQQLSKL **G** RNEE TPGSFSADDD ELER AGLGKIGAFIKKAYAIYKAKAA **G** RNEQ TPATVSANDD EEAR KIKWFKAMKSIAKYVAKKQLKKHLGGEN *
	m.2734	<EEARIWLTALKFIGKNLGKHFAKQQLSKL **G** RSED MTVNDD EEAR IWLTALKFIGKNLGKHFAKQQLSKL **G** RSED MSANDD EEAR IWLTALKFIGKNLGKHLAKQQLSKL **G** RNEE TPGSFSADDD ELER AGLGKIGAFIKKAYAIYKAKAA **G** RNEQ TPATVSANDD EEAR KIKWFKAMKSIAKYVAKKQLKKHLGGEN *
	m.2795	<EEARIWLTALKFIGKNLGKHFAKQQLSKL **G** RSED MTVNDD EEAR IWLTALKFLGKNLGLKFIGKNLGKHFAKQQLSKL **G** RSED MSANDD EEAR IWLTALKFIGKNLGKHLAKQQLSKL **G** RNEE TPGSFSADDD ELER AGLGKIGAFIKKAYAIYKAKAA **G** RNEQ TPATVSANDD EEAR KIKWFKAMKSIAKYVAKKQLKKHLGGEN *
	m.2773	<EEARGRLQAFLAKMKEIAAQTL **G** REES VSANED EEAR SKWKAFLAKMKEIASEAL **G** REEN LSANED EEER MVWLLPLKFLASHVAMEQLSKLGSKIATKL **G** RNEQ IPVISANED EEER MVWLLPLKFLASHVAMEQLSKLGSKIATKL **G** RNEE TPVSFFADDD GEER AGLGKIGALIKKAYAIYKAKAA **G** RNEQ TPATVSAND DEEAR KIKWFKAMKSIAKYVAKKQLKKHLGGEN *

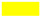
 Mature region; 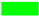 PQM; 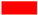 iPQM; 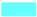 Spacer or linker; 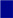 C-terminal amidation; < Signal peptide and propeptide; > Truncated C-terminal sequence; * End of the sequence.

**Table 2:** Mature sequences of the linear peptides predicted in this study. The nomenclature of the peptides was based on the method proposed by King et al. [35], wherein ‘M’ refers to the action target of the peptide (membrane), followed by a generic name (lycotoxin) of similar peptides. ‘Lp’ refers to the first two characters of the species name (*Lycosa poonaensis*), followed by a number (n) to distinguish similar toxins.

Name	Sequence ^1^	Detection in the Venom ^2^
M-lycotoxin-Lp1a	**MVWLLPLKFLASHVAMEQLSKLGSKIATKL-NH_2_**	F
M-lcotoxin-Lp1b	**VIWLPALKFLASHIAMEQLSKL-NH_2_**	S
M-lcotoxin-Lp2 ^3^	**GRLQAFLAKMKEIAAQTL-NH_2_**	F
M-lcotoxin-Lp3a	**IWLTALKFIGKNLGKHFAKQQLSKL-NH_2_**	F
M-lcotoxin-Lp3b	**IWLTALKFLGKNLGKHFAKQQLAKL-NH_2_**	n.d.
M-lcotoxin-Lp3c	**IWLTALKFIGKNLGKHLAKQQLSKL-NH_2_**	S
M-lcotoxin-Lp3d	**IWLTALNKFIGKNLGKHFAKQQLSKL-NH_2_**	S
M-lcotoxin-Lp3e	**IWLTALKFLGKNLGLKFIGKNLGKHFAKQQLSKL-NH_2_**	n.d.
M-lcotoxin-Lp3f	**I W L T AL K F LG K N LGL K F IG K N LG KH **	n.d.
M-lcotoxin-Lp3g	**I W L T AL K F IG K N LLV NS I K I F R **	n.d.
M-lcotoxin-Lp3h	**I W L T AL K Q L S K LG R T **	n.d.
M-lcotoxin-Lp3i	**IWLTALNNASEAL-NH_2_**	n.d.
M-lcotoxin-Lp3j	**IWLTALNKFL-NH_2_**	n.d.
M-lcotoxin-Lp4	** K I K WF K AM K S IA K VA KK Q L KKH LGG E N **	F
M-lcotoxin-Lp5a	**AGLGKIGAFIKKAAIKAKAA-NH_2_**	F
M-lcotoxin-Lp5b	**AGLGKIGALIKKAAIKAKAA-NH_2_**	F
M-lcotoxin-Lp6	**SKWKAFLAKMKEIASEAL-NH_2_**	F

^1^ Aliphatic hydrophobic residues are shown in black, aromatic hydrophobic residues in grey, basic residues in blue, acidic residues in red, neutral polar residues in pink, and proline in green. ^2^ F, detected as full length; S, detected as short length; n.d. not detected. ^3^ This peptide was renamed from lyp1987.

**Table 3:** Physicochemical properties of the peptides synthesized in this study.

Name	Number of Residues	Molecular Mass	Net Charge	Hydrophobic Moment ^1^	Hydrophobicity ^1^
M-lycotoxin-Lp1a	30	3350.9	+4	0.416	0.358
M-lycotoxin-Lp1b	22	2506.4	+2	0.176	0.838
M-lycotoxin-Lp3a	25	2880.7	+6	0.538	0.510
M-lycotoxin-Lp4	28	3271.9	+8	0.455	0.316
M-lycotoxin-Lp5a	22	2251.3	+6	0.427	0.449
M-lycotoxin-Lp5b	22	2217.3	+6	0.425	0.444
M-lycotoxin-Lp6	18	2049.1	+3	0.482	0.355

^1^ Calculated using the online software HeliQuest (https://heliquest.ipmc.cnrs.fr, accessed on 14 July 2022). Hydrophobicity was calculated in the region with the highest hydrophobic moment.

**Table 4:** Estimation of secondary structures of the synthesized peptides.

Peptides	Solvents ^1^	Percentage of Secondary Structure ^2^			
		α-Helix	β-Sheet	Turns	Unordered
M-lycotoxin-Lp1a	I	15	26	25	35
	II	61	10	11	19
M-lycotoxin-Lp1b	I	10	31	26	33
	II	57	9	12	22
M-lycotoxin-Lp3a	I	11	29	26	34
	II	59	16	8	16
M-lycotoxin-Lp4	I	8	33	26	33
	II	51	11	13	23
M-lycotoxin-Lp5a	I	15	23	28	33
	II	55	17	10	18
M-lycotoxin-Lp5b	I	7	33	25	34
	II	51	14	11	23
M-lycotoxin-Lp6	I	20	22	25	33
	II	55	14	8	22

^1^ Solvent I indicates a 0.2 M phosphate buffer (pH 7.0), and solvent II indicates 50% TFE in a 0.2 M phosphate buffer (pH 7.0). ^2^ Calculated using the DichroWeb server with the CDSSTR method and reference dataset 4.

**Table 5:** Biological activities of the synthesized peptides.

Peptide	Antibacterial Activity MIC (µM)			Insect Toxicity ^1^ PD_50_ (µg/g Weight)	Hemolysis EC_50_ (µM)
	*E. coli*	*S. aureus*	*B. subtilis*		
M-lycotoxin-Lp1a	6.2–12.5	12.5–25	12.5–25	35 (5.92–20.4)	>100
M-lycotoxin-Lp1a(9-30) ^2^	>100	>100	100–200	>1000	>100
M-lycotoxin-Lp1b	50–100	3.1–6.2	6.2–12.5	31 (13.7–68.2)	>100
M-lycotoxin-Lp3a	0.8–1.6	0.4–0.8	0.8–1.6	70 (46.4–149)	>100
M-lycotoxin-Lp4	0.4–0.8	1.5–3.1	3.1–6.2	10 (5.8–17.1)	>100
M-lycotoxin-Lp5a	1.25–2.5	50–100	1.25–2.5	50 (18.3–138)	>100
M-lycotoxin-Lp5b	1.25–2.5	50–100	1.25–2.5	44 (12.2–160)	>100
M-lycotoxin-Lp6	5–12.5	>100	5–12.5	94 (53.4–168)	>100

^1^ Values in parentheses indicate 95% confidence intervals. ^2^ Data from Ref. [13].

## Data Availability

The data underlying this article will be shared on reasonable request to the corresponding author.

## Supplementary Materials

The following supporting information can be downloaded at: https://www.mdpi.com/article/10.3390/toxins14120854/s1, Figure S1: High-resolution LC/MS analysis of the *L poonaensis* venom. Figure S2: CD spectra of linear peptides synthesized in this study; Table S1: List of the sequences of precursors of peptides and proteins identified by transcriptomic analysis of the *L. poonaensis* spider venom gland.
