# Supplementary material for: Molecular Diversity of Linear Peptides Revealed by Transcriptomic Analysis of the Venom Gland of the Spider Lycosa poonaensis
Source: Toxins (Basel). 2022 Dec 3;14(12):854. doi: 10.3390/toxins14120854 (PMC9788040; doi:10.3390/toxins14120854)
Supplement: Supplementary file 1 [file toxins-14-00854-s001.zip › FigureS1.pdf]

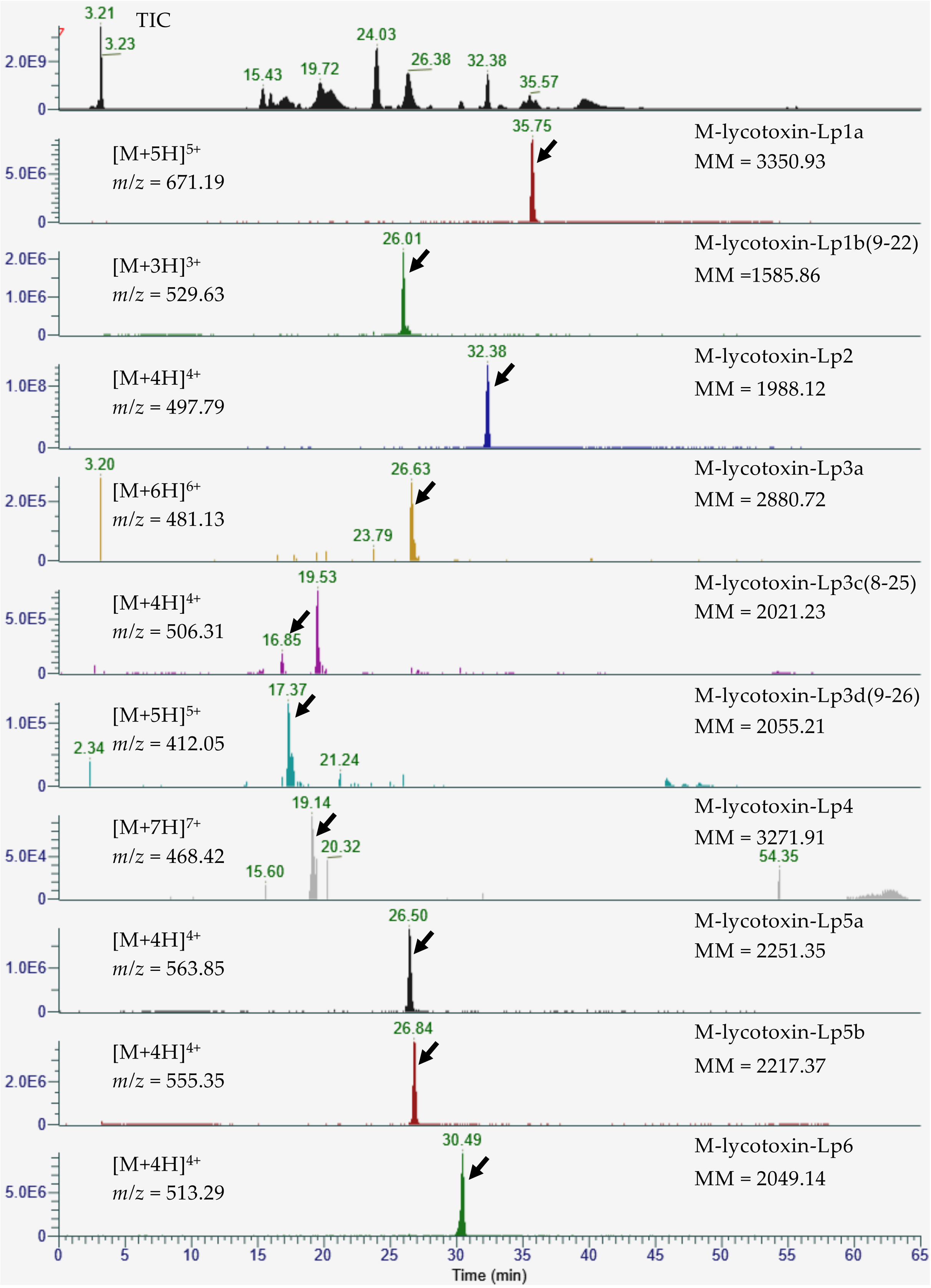

Figure S1 High-resolution LC/MS analysis of the *L poonaensis* venom. Arrows indicate peaks corresponding to linear peptides.
