# Supplementary material for: Molecular Diversity of Linear Peptides Revealed by Transcriptomic Analysis of the Venom Gland of the Spider Lycosa poonaensis
Source: Toxins (Basel). 2022 Dec 3;14(12):854. doi: 10.3390/toxins14120854 (PMC9788040; doi:10.3390/toxins14120854)
Supplement: Supplementary file 1 [file toxins-14-00854-s001.zip › FigureS2.pdf]

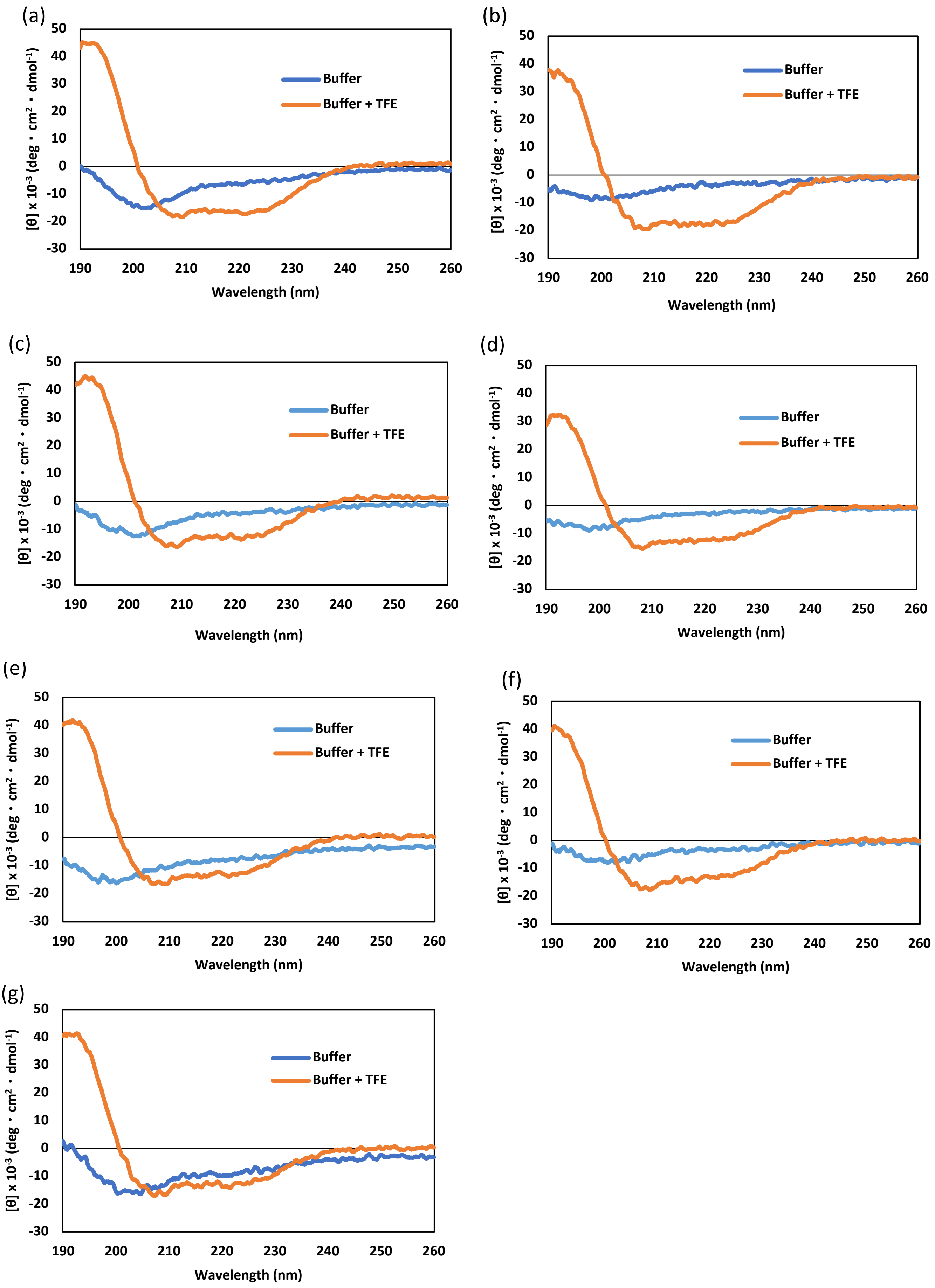

Figure S2 CD spectra of linear peptides synthesized in this study. (a) M-lycotoxin-Lp1a, (b) M-lycotoxin-Lp1b, (c) M-lycotoxin-Lp3a, (d) M-lycotoxin-Lp4, (e) M-lycotoxin-Lp5a, (f) M-lycotoxin-Lp5b, and (g) M-lycotoxin-Lp6. Spectra were obtained in 0.2 M phosphate buffer at pH 7.0 (dotted line) and in 50% TFE in 0.2 M phosphate buffer at pH 7.0 (solid line).
