# Supplementary material for: Molecular Diversity of Linear Peptides Revealed by Transcriptomic Analysis of the Venom Gland of the Spider Lycosa poonaensis
Source: Toxins (Basel). 2022 Dec 3;14(12):854. doi: 10.3390/toxins14120854 (PMC9788040; doi:10.3390/toxins14120854)
Supplement: Supplementary file 1 [file toxins-14-00854-s001.zip › TableS1.pdf]

Table S1. List of the sequences of precursors of peptides and proteins identified by transcriptomic analysis of the *L. poonaensis* spider venom gland

(1) Neurotoxins

Name	Sequence	Similar peptide (Accession No., species)
m.59186	MSSGPSLSLSDTAHFRKSCLEIGEVCDGNANDCQCCRSNGFCHCSWIFNHCTCQVGDSSKSYGVCLWKQKNCPNKPGMCTKPCTNRRRCKNRSG	ω-ctenitoxin-Pn3a (P81790, *Phoneutria nigriventer*)
m.1192	MKTSTVFGLCAIVVLMLTIADLSGADEMNSQDAPEERGYCAEKGIRCDDIHCCTGLKCKCNDSGYNCVCRKK	Purotoxin-1 (P86269, *Alopecosa marikovskyi*)
m.38262	MGRWIFAIFLGITLLTQVLLPSAYMATSDADTPAVDDYADVARLLYFARKRSCIKRGSSCDHRPNDCCDNSSCRCNLWGTNCRCQRMGLFQKWGK	U8-agatoxin-Ao1a (Q5Y4U4, *Agelena orientalis*)
m.218293	MKYFLKPLVVTVLLLLCYAVMTNAYVMRDSSLDSYQEPNYEALRQYLLSTRNLNNIPNRRSCIRRGSSCDHRPSDCCFNSSCRCNLWGTNCRCQRAGLFQKWGK	U8-agatoxin-Ao1a (Q5Y4U4, *Agelena orientalis*)
m.9	MSPKVQALLLFVGLITFLVVHAEELSEIEESERACAEEHKSCNWENKPCCDKMSCHCSAAFTNCKCQKGVIRKIIDWIGGK	U16-lycotoxin-Ls1a (B6DD52, *Lycosa singoriensis*)
m.11889	MSPKMQVLLLTIWLFMFLAVHSHEELSESAESERSCANEYQSCDWYNRPCCDNISCVCSWIGTNCECKKGIIRTIRDWINGK	U16-lycotoxin-Ls1a (B6DD52, *Lycosa singoriensis*)
m.16586	MIRYVLISALLVVAVYSFTFEDENEDVFLEEAKEELDPEEERRIALPPGAVCNGHKSDCQCFGAKYKCSCPWLWRFRRSAKCHCKKGWAWTAIKKRSCRNRYQWSG	U2-lycotoxin-Ls1a (B6DD29, *Lycosa singoriensis*)
m.30919	MKILFVLISILYSVHCFTSEEIVGSEHLANELEAVEDINSEQNVALESTREEERSCADMGQDCADDCDCCLNIARCNCWFGKYFCSCTFGNYQTCQAKRGKCTRNRPQSCPKSNINHKKG	U13-lycotoxin-Ls1c (B6DD20, *Lycosa singoriensis*)
m.8162	MKIAVIFGVLLVTLFSYSSAEMFDDFEQADEADELLSLIEEQTRAKECTPRFNDCTNDRHSCCRGELFKDVCTCFYPENGGNEFCTCQQPKHFKYIEKGTDKIKKIGSKIKSWFG	U3-lycotoxin-Ls1a (B6DCQ5, *Lycosa singoriensis*)
m.6715	MSTFTQCKRKVIFNKLFSKQIPLIFPFYRAKACTPRFRDCSHDRHSCCRSSLFKDVCTCFYPENGDKKEVCSCQQPKHLKYMEKATDKIKYLIG	LSTX-D6 (B6DCU5, *Lycosa singoriensis*)
m.6722	MKVFVVVALLVTLISYSSSEGIEDLEADDFLSLMANEQPRAKACTPRFRDCSHDRHSCCRSSLFKDVCTCFYPENGDKKEVCSCQQPKHLKYMEKATDKIKYLIG	LSTX-D6 (B6DCU5, *Lycosa singoriensis*)
m.6716	MNFLKGNINTFRNDCIPKHHECTSNKHGCCRGKIFKYKCQCTTVVDQSGEQAERCFCGTATHHKAVELMAGFGKKLFG	U1-lycotoxin-Ls1ee (B6DCN6, *Lycosa singoriensis*)
m.103490	MANEQPRSDCIPKHHECTSNKHGCCRGKFFKYKCQCITVVDQSGEQAERCFCGTATHHKAVELMAGIGKKLFG	U1-lycotoxin-Ls1e (B6DCJ5, *Lycosa singoriensis*)
m.6709	MKVFVVVALLVTLISYSSSEGIEDLEADDFLSLMANEQPRNDCIPKHHECTSNKHGCCRGKIFKYKCQCTTVVDQSGEQAERCFCGTATHHKAVELMAGFGKKLFG	U1-lycotoxin-Ls1e (B6DCJ5, *Lycosa singoriensis*)
m.6148	MKYQILFGLVFLTLLSYCSSEFEDAGLFMDEEMVEADDPNAIARKDDNENCIAKHHECTSNRHGCCRGKLFKYKCQCVKIVNAQKEETERCACITPGLHKAVEFVLQLFKKAIA	U5-lycotoxin-Ls1a (B6DCV0, *Lycosa singoriensis*)
m.69253	MKVLVLFTVLFLTLFSYSSTEAIHEFDSNAEEDMLSVFACEQVRAKACSPRLHDCSDDRHSCCRGEKYKVICNCYDLEGEDKSEVSTREQPKSEKNIVKLDEKSKTVV	U4-lycotoxin-Ls1a (B6DCT6, *Lycosa singoriensis*)
m.157117	MKVLVIFSVLFITLFSYSSTEAIDEFDSNAEEDMLSVLASEQERAKACTPRLHDCSDERHSWCRGEMFKDVCYCFYPEGEDKTEVCSCQQPKSHKYNEKVVDKTNTLVG	U4-lycotoxin-Ls1a (B6DCT6, *Lycosa singoriensis*)
m.190400	MKLLFFTGLFLLVVVNLIKAEAEAENERAACIRLERQCTKTPGNCCSGLKCECYQRFEKGVAKGKRCWCIEKDVTYKNKK	U6-lycotoxin-Ls1d (B6DCV4, *Lycosa singoriensis*)
m.2157	MKILFFTLSLLLVASFIEVEAKSKGSCLTYAEVCTKNASGCCSDLRCDCYIRYEEGVEKGEKCWCNYKDVIYKKA	U7-lycotoxin-Ls1c (B6DCW9, *Lycosa singoriensis*)
m.2159	MKIVLFAVIAVLLTVSLIEIEAQSKGVCLPYAAVCKTNASRCCSGLTCDCYLRFEEGVQKGEKCWCIHKGVTYTKE	U7-lycotoxin-Ls1c (B6DCW9, *Lycosa singoriensis*)
m.10500	MKLIIFTGLVIFAIANAIEAEAENERACIAEYGECLKAPGNCCSNLACDCYGRFKNGKEIGRNCFCLQKGVIYKREK	U8-lycotoxin-Ls1s (B6DCX9, *Lycosa singoriensis*)
m.10497	MKLIIFTVLVLFSIVSLIKAEAESERACVAQYGECLKEPGNCCSNLVCDCYGRFKNGKEIGRNCFCLEKGIIYKREK	U8-lycotoxin-Ls1s (B6DCX9, *Lycosa singoriensis*)
m.10498	MKLIIFTGLVIFAIANAIEAEAENERACIAEYGECLKAPGNCCSNLACDCYGRFKNGKEIGRNCFCLEKGIIYKREK	U8-lycotoxin-Ls1s (B6DCX9, *Lycosa singoriensis*)
m.3722	MKLGIFLLFSLVVLAVASESIEENINDNLPEQQRACADLNEKCTEGDDCSCCGERGRCDCNWPGKPGCYCMRGGPFDLIAKKFKC	U12-lycotoxin-Ls1a (B6DD13, *Lycosa singoriensis*)

(2) Cytolytic linear peptides

Name	Sequence	Similar peptide (Accession No., Species)
m.2649	MTVNDDEEARIWLTALKFIGKNLGKHFAKQQLSKLGRSEDMSEMTMKKQESG	LyeTx I (C0HJU9, *Lycosa erythrognatha*)
m.2799	MSANDDEEARIWLTALKFLGKNLGKHFAKQQLAKLGRSEDISENLSADSFQSLEEVKTCLQMTMKKQESG	LyeTx I (C0HJU9, *Lycosa erythrognatha*)
m.2698	MSANDDEEARIWLTALKFIGKNLGKHFAKQQLSKLGRSEDMTVNDDEEARIWLTALKFIGKNLGKHFAKQQLSKLGRSEDMSECLQMTMNKQESG	LyeTx I (C0HJU9, *Lycosa erythrognatha*)
m.2695	MSANDDEEARIWLTALKFIGKNLGKHFAKQQLSKLGRSEDMTVNDDEEARIWLTALKFLGKNLGLKFIGKNLGKHFAKQQLSKLGRSEDMSECLQMTMNKQESG	LyeTx I (C0HJU9, *Lycosa erythrognatha*)
m.2781	MTKKKQESWLTALRIWLTALKFLGKNLGKNLGKHLAKQQLAKLGRSEDISENDDEEARIWLTALKFLGKNLGKHFAKQQLAKLGRSEDISENLSADDDEEARIWLTALKFIGKNLLVNSIKIFR	LyeTx I (C0HJU9, *Lycosa erythrognatha*)
m.2690	MSANDDEEARIWLTALKFLGKNLGKHRISQNSSLQSLEEVKTSLKTCLQSQNNSFQSLEEVKIMSANDDEEARIWLTALKFIGKNLGKHFAKQQLSKLGRSEDMTVNDDEEARIWLTALKFLGKNLGLKFIGKNLGKH	LyeTx I (C0HJU9, *Lycosa erythrognatha*)
m.2667	MKEIAAQTLGREESLSENENEEARGRLQAFLAKMKEIAAQTLGREENLFANEEERVIWLPALKFLASHIAMEQLSKLGRNEQTPEEARIWLTALKFIGKNLGKHFAKQQLSKLGRNEQTPEEARIWLTALKQLSKLGRT	LyeTx I (C0HJU9, *Lycosa erythrognatha*)
m.2650	MKEIAAQTLGREESLSENENEEARGRLQAFLAKMKEIAAQTLGREENLFANEEERVIWLPALKFLASHIAMEQLSKLGRNEQTPEEARIWLTALNKFIGKNLGKHFAKQQLSKLGRNEQTPEEARIWLTALKQLSKLGRT	LyeTx I (C0HJU9, *Lycosa erythrognatha*)
m.2660	MKEIAAQTLGREESLSENENEEARGRLQAFLAKMKEIAAQTLGREENLFANEEERVIWLPALKFLASHIAMEQLSKLGRNEQTPEEARIWLTALNKFLG	M-lycotoxin-Ls3b (P0C2U7, *Lycosa erythrognatha*)
m.2706	MKEEIAAQTLGREESLSENENEEARGRLQAFLAKMKEIAAQTLGREENLFANEEERVIWLPALKFLASHIAMEQLSKLGRNEQTPEEARIWLTALNNASEALGREEIWGFCK	M-lycotoxin-Ls3b (P0C2U7, *Lycosa erythrognatha*)
m.2678	MKEIAAQTLGREESVSANEDEEARSKWKAFLAKMKEIASEALGREEGVSANEDEEARGRLQAFLAKMKEIAAQTLGREDSLSANEDEEARGRLQAFLAKMKEIAAQTLGREESLSENENEEARGRLQAFLAKMKEIAAQTLGREESVSANEDEEARSKWKAFLAKMKEIASEALGREENLSANEDEEGGFCK	M-lycotoxin-Ls3b (P0C2U7, *Lycosa erythrognatha*)
m.2715	MSANDDEEARIWLTALKFIGKNLARIWLTALKFLGKNLGLKFIGKNLGKHFAKQQLSKLGRSEEARIWLTALKFIGKNLGKHLAKQQLSKLGRNEETPGSFSADDDELERAGLGKIGAFIKKAYAIYKAKAAGRNEQTPATVSANDDEEARKIKWFKAMKSIAKYVAKKQLKKHLGGEN	M-lycotoxin-Hc2a (P61508, *Hogna carolinensis*)
m.2734	MSANDDEEARIWLTALKFIGKNLGKHFAKQQLSKLGRSEDMTVNDDEEARIWLTALKFIGKNLGKHFAKQQLSKLGRSEDMSANDDEEARIWLTALKFIGKNLGKHLAKQQLSKLGRNEETPGSFSADDDELERAGLGKIGAFIKKAYAIYKAKAAGRNEQTPATVSANDDEEARKIKWFKAMKSIAKYVAKKQLKKHLGGEN	M-lycotoxin-Hc2a (P61508, *Hogna carolinensis*)
m.2795	MSANDDEEARIWLTALKFIGKNLGKHFAKQQLSKLGRSEDMTVNDDEEARIWLTALKFLGKNLGLKFIGKNLGKHFAKQQLSKLGRSEDMSANDDEEARIWLTALKFIGKNLGKHLAKQQLSKLGRNEETPGSFSADDDELERAGLGKIGAFIKKAYAIYKAKAAGRNEQTPATVSANDDEEARKIKWFKAMKSIAKYVAKKQLKKHLGGEN	M-lycotoxin-Hc2a (P61508, *Hogna carolinensis*)
m.2773	MKEIASLQMRMKKREIAAQTLGREESLSENENEEARGRLQAFLAKMKEIAAQTLGREESVSANEDEEARSKWKAFLAKMKEIASEALGREENLSANEDEEERMVWLLPLKFLASHVAMEQLSKLGSKIATKLGRNEQIPVISANEDEEERMVWLLPLKFLASHVAMEQLSKLGSKIATKLGRNEETPVSFFADDDGEERAGLGKIGALIKKAYAIYKAKAAGRNEQTPATVSANDDEEARKIKWFKAMKSIAKYVAKKQLKKHLGGEN	M-lycotoxin-Hc2a (P61508, *Hogna carolinensis*)

(3) Protease inhibitor

Name	Sequence	Similar peptide (Accession No., species)
m.201956	MNFQFVLLCMTSVVVVQYAVAFKLIRSKRSTNVPSRCLQDPDSGMCLAYFLNYYYNPSSGECETFVYGGCQGNDNNFWTKDDCEAICKPKTNETEEINSL LLESLESLIEGKDVCEYPPETGECRALFIRYYYDGEKCSPFVYGGCDGNPNNFETEVECMERCGPKSEDESEENEV	U19-barytoxin-Tl1a (W4VSH9, *Trittame loki*)
m.221752	MKNISIFSVVLTLSFSLAVSFTYPRSRRSSDDEDSSSRCQLPPVTGLCRAYFLMYYHNATTNTCNRFVFGGCIGNGNKFWTGEECVEACGGTIDDTMPIQS FHYDYDFEDYDDPCDKPLVVGHCDADIPRFYFDGKECTAFLYGGCGGGNHNNFPTKEKCLEKCGYRILDEENEIENTE	U19-barytoxin-Tl1a (W4VSH9, *Trittame loki*)
m.248055	MEGNMLMVLLVASTIGLALSNPLVSRPNYDNIPQCLQPPASGMCLAYFPSYYYNPSKETCDTFIYGGCQGNANRFFSFDECMAKCGGFKQEPNDSFKLIL ENGEDQNPSASEQQVEDICSLSTEMGPCRAMMPRFYFNGDKCEEFIYGGCEGNANNFKTIEECQQRCGSTSSSEEVEADNSEETEKASEEAVAEEASVE SAEESDSKAEN	U19-barytoxin-Tl1a (W4VSH9, *Trittame loki*)
m.250351	MKNEFLILFLIIGLFCPIYGILESDRLKRLIRIDLNNPLAKDVCDYPLDIGRCFGAKQRYYFNGDTCEEFTYKGCLGNANNFRTKEECMLKCAHKMEGGSGET EEEEHFSFSLFG	kappaPI-theraphotoxin-Hs1a (P0DJ77, *Cyriopagopus schmidti*)
m.41663	MDSKIIAVFLLLVLSTCVLSEKYCPTTRNPYCKKMNIRNNCCKDSDCSSGYCCSEPCGNFCHAPSSIPGGKRVNPNASCKLGYVYR	U14-lycotoxin-Ls1b (B6DD37, *Lycosa singoriensis*)
m.1940	MNSKIFAVLLLLALSACALSEKYCPTPRNTSCKKQNIRNDCCKDSDCTSNAFCCAQPCGNFCIAPSDNPGGRRVDPNASCKLGYVY	U14-lycotoxin-Ls1b (B6DD37, *Lycosa singoriensis*)
m.41662	MPHVLCEFYINKRMVVSNHYIQLSEKELICKMNSKIFAVLFLLALLTCVLSDQYCPKSRLTPCKKMNIRIDCCKDEDCTGGSWCCATPCGNFCKYPINRPGG QRAAGGENCKIGYVYLK	U15-lycotoxin-Ls1d (B6DD42, *Lycosa singoriensis*)
m.11922	MNSHILFVLLVLGIATCVLAGDIELRINICSNELKANRKKCNENCKRLSFQDFGIKKEDRTTLFCFLHQIKPLR	U20-lycotoxin-Ls1c (B6DD62, *Lycosa singoriensis*)
m.240217	MKPTYLFICVISLVYLVSAGRSQFCPVREPIFCIRRQDLCCSHNECSVGELCCEENCGNVCRLAVPRRTSGIEYRYNPNCINESL	U7-agatoxin-Ao1a (Q5Y4V9, *Agelena orientalis*)
m.11914	MNSHILFVLLVLGIATCVLAGGFCPKSRHPHCNLSYKINDCCAQSDCRFGSVCCVEGCGNVCRAESDTPIGEKFVDGSECKLGHVFPKRWYEFW	U20-lycotoxin-Ls1a (B6DD60, *Lycosa singoriensis*)
m.204789	MMFKILLVAIFIVLAESTERKTDCQEHREREAKSKASLPMRLIPECDENGDYKPLQCFKDSDFCACWDKQGNPVTQPSKTIKQCDCLVQKHEVEKKGLRGA FKPSCSEDGRYQKQQCHGSTGFCWCAHPETGEKTSEETRGRPNC	U24-ctenitoxin-Pn1a (P84032, *Phoneutria nigriventer*)
m.217252	MWIPIFLLALAVPTIRSERKCGECVPEKCQPPSEECLAGLVKDLCGCCYVCGRREGELCDGDFLPIPYRNRGYGPCGEHLECRPRTDLAPGDPPEAMCVC LKTETLCGSDGNTYDNECQLTEARYKQREQILKAMHRGPCRSAPKITSPPEEASNYTGGNIAMSCEATGWPIPVFEWRVDIGDGNTIPLPSDDPKVSVQS RGGPSKYEVTSWLQLLSIQPKDDATYWCIAKNDEGESSAAARVVVLDFRGSKTEDNGRINDL	IGFBP-rP1 (G4V4G1, *Cupiennius salei*)

(4) TCTP family

Name	Sequence	Similar peptide (Accession No., species)
m.7640	MIIFKDLITGDEMFTDSSKYKLIDECLYEVECRHVQRRQGDIQLEGANPSQEEADEGTDESVESGLDLVLNQRLVETGFTKNDYKNYLKTYTKSLQDKWKE MGKNDTEIAEAKSKITEAVKKVLPKLADMQFFMGESSNPDGLIALLEYRENPGGDETPIMIFFKHGLDEEKV	Translationally controlled tumor protein homolog (G3LU44, *Loxosceles intermedia*)
m.24068	MRIFKDIITGDEMFTDSSKYKLIDDCLYEVECRHVQRRLGDVQLDGANPSQEEADEGTEEVVESGLDLVLNQRLVETVFSKNDYKGYLKLYTKSLQDKWKE MGKSDSEIADAKSKFTEAVKKILPKIGDLQFFMGESSDPDGLIALLEYRENESGNETPIMMFFKHGLEEEKV	Translationally controlled tumor protein homolog (G3LU44, *Loxosceles intermedia*)

(5) Non-toxic peptide

Name	Sequence	Similar peptide (Accession No., species)
m.106956	MKCIIALTILATLVVAIQGKFCERSSDCGEGQCCTGSSFNRHCQELSDNGRPCQRPNDYDSYKTGCPCKEGLICSVINYCQEP	U19-ctenitoxin-Pn1a (P83997, *Phoneutria nigriventer*)
m.7591	MRTMKCIISLTIVATLMCLVQGQDQSCGEVYCGEGQCCSGSFYARHCRDYSNDGEPCERPNKYNSYKTACPCKEGMFCNVINRCQKYE	U19-ctenitoxin-Pn1a (P83997, *Phoneutria nigriventer*)

(6) Peptidase

Name	Sequence	Similar protein (Accession No., species)
m.1011	MDTSVSPCDDFYQYACGRWSKHHELPSDRSYYNTFEVLKDQLKAQLKDLLEEPVTDDDSNATIKAKNLYASCMNEEVIEELKEKPLVLLLDDLGGWPVTS<br>SNWTEENFDWVYLIATLQQYSNNILISQWVGPDGRNSSMHIIQIDQADLGLPSREYYVQGTQQLDAYGRYMVEIAQLLGASPEKAEEEMKEVLEFEIEIANF<br>TIPREERRNYTAMYHKLTLAELQVRIPLVNWTLFFNVAMPLELTDDEEIVIYAITYVERMTDFLVNTPKRVVANYILWRFVYKFVSNLDKRFQAKQQEYYSAL<br>YGTQSIPPRWKTCTVYTNKIMGTAVGSLFVKTHFNERSKETAEEMIENIKTAFLELLEEVDWMDTETRSEARQKALLMTEKIGFPDYIMDPQELDKDYDGIEI<br>KPDRYFDNVLLSLKQYSIREQMKLRTLVDRTQWVSSPAVVNAFYTRPKNFITIPAGILQPPLYHQNYIRSLSYGGIGVVIGHEITHGFDDKGRQFDHRGNLK<br>QWWDSEALIKFQSKADCMIKQYGQYMMTDINMKVNGINTQGENIADNGGVKQSFKAYKSWASRNSEEPLLPGLNLTHDQLFFLNYAQIWCGSMRPEAAI<br>NMIRTGVHSPGKYRVIGALSNSKDFSEAYGCPLGSPMNPEKKCHVW	Neprilysin-1 (W4VS99, *Trittame loki*)
m.15154	MVSKGGCLGWLKKRTNLEKTLLSLSSLLLLTLLLMILIGITYQGKKTKNSPENEEECWSTECIQTAGDILKKIEPEADSCGNFYEYACGTYTKNLGQTISVPKQ<br>STDSVYFTLKRLMESKKNLDVRNLKDVRSFYDTCVKFGSHWQDLKETGDSLNNLMLDLGVGTWPIIDSLYDTSTFSRDKTFASLILLDVPVAFQMEIVPDQ<br>QISGSFLVKISPGSPRESGRSPSDIRGDVHLRSSVLTSFLLLGANAKITDVNDIIAADSSFAAIEQDKNIVCDDVLLSPQEAPSELKRLIPEIDWKIILSEIRNVTG<br>LVRPFAVELHCKQKIRDYVVHLNDLTVRSEANYLGWRFFSKFVEHVVPTFEKSQFGTDDVVPRWRICLELIEKFSPTVLVDLLAKEIINKEVIAQTERISDSSL<br>KAAKRLVSRTQWLTNNQRSRIQEKVSRFRTRYPFKRTAADGDSGKLKIPQVNVNNFTAVIVRQQRKWVIQRFRRLSHEADNVDWIESRVPFLTARSDSVL<br>SDSVLYVYFDKIRTPYIQLKGSQWLNYGGFGVTVAREISRLLLSSSVGQGENTLLWNLWSNSSNTSKCLITSMAKRMGIHEEVLNALADEKLLQNLFLDQSA<br>IEIAYETAKMTAGSSKSLPGLELNDEKMFFLSYAQTQCEADLQVQKSKNIPPIQNRLNSVLQNFDSFNEVFSCASSSHCQIWA	Neprilysin-1 (W4VS99, *Trittame loki*)
m.19936	MRRADSTLKARDRYEKHSLTKFRNETGICYSPSCISEASNLLKSINMSVDPCSDFYSFACGNWIKNAYLVSTLESQFTKLTRIIHLRVKSILEDLEKEDNLPDH<br>KMKTSDFYKACMDRDHRNEIGATEFLNDLKKFGGWPLIEEEWDAANFNWSNVMIQLFKNGYISNMLFTVKLEIDAKNTSRSIIAVMQPSLGLKNKLYYIIPD<br>GQIATSYKKAIFEVAKHLHPNANLVKKTSMDAIEKAFELESKIADLIKEVNMRYPDQFYNIMTLEELENFAPKIPWRNLLKHLLPPETILTNEERILVTVPSMIKG<br>LNKILEEMSTEQGKRILSNYMFYRTAFFAASNLNTEFYNLIKGIFNTDSVANNQLWEICVKDTMDLFPLPLTSSYVAKYLKNNTKELLEVMFDDIKNALVEDIK<br>NAAWLDTETKNYALFKVKKMKAAIAYKEEIMKDENLNSFYKEVNVTDNHFSNVKQLIAARQQRNMQGLHKKRDRFRWTDVNNLLTANAYYQPSSNAIIIPV<br>GVLHDTIFAAERPNYINYGSLGMIVGHELTHAFDSIGSLYDADGSYRMWWKKESWKKFEERVKCYKEQYDKFYLSEADSNVNGTLTLGENIADNGGLQTA<br>YYAYNLLLKRLGSEKSLPGLPYTERQMFWISFANVWCRKQTEESIQYGMIYSVHSPSRFRIIGSLSNMKEFSEDFNCKLNSTMNPKSKCLLWK	Neprilysin-1 (W4VS99, *Trittame loki*)
m.9580	MAGNLAVAFRNVTAQKCAWWNRRSPCEKFLFILCILLLSTCAVLIIVNLITEKRLRVAEDIVSGICHSEECIKAAARILERLDTSTKPCDDFYHFACGNFLHRHT<br>VPDDHYLRSTMQTMQDDLYVTLKRLIEHPQSSNDSEAIIKVKKLYTSCMNTSSIEDGSVKVLQDLLTNWGIGEWPILIPRWNKTVDMEWRLAMLHIHEVKPF<br>FSTFVAPDDRNSSVYLLHINAGGPFLNPQYFLNTSDPDYVRYVLSYKNLIFETVRLLGVQESDAKKDIEEMLEFEVEFANISQDDPFDSINETSQLDDDFVFN<br>KINVSVLEDMIPEIKWNVLIEYIFDYIGLSTEKIDISFVVHCEKYLRHLVALLNRTPPKTAINYLTWRFVMKYMPYLDIHFRRLYYDFRREVPVRAEERTFFARW<br>KECVHIATEGFGMALASLYVKDEFNEEMENEIKFLISSLKEAFIKSIHKQTWLDHDTKVLCEEKVSAMGNKIGFPRYILDPFQLDADYVGLDVTEDHFLDNILK<br>INRYEETKDLSKITRTVDKERDWFVQPLVVNAYYEGSGNTVIFPVGILRRPIFTPRRPKYLNYGMLGVIIGHEITHGFDNNGRKYDKAGNFTQWWTDEIVEK<br>FKEQVSCYIEQYSQLPIDMVGQNVNGNQTLDDNICDNSGLAQAYRAYRHYVSKYGEEPRLPGISYSNTQVFFLQYAQLWCEILSKEANERFIKDSHSPGKY<br>RANIPLMNSPEFSSAFNCPTGSPMNPVKKCRLWA	Neprilysin-1 (W4VS99, *Trittame loki*)
m.19952	MFTSQYMNFDEKSRLYPERGPGKTFPVTTPLERTLLIIIAIVVLIGIGLSIGIVSLKKARDRYEKHSLTKFRNETGICYSPSCISEASNLLKSINMSVDPCSDFYS<br>FACGNWIKNAYLVSTLESQFTKLTRIIHLRVKSILEDLEKEDNLPDHKMKTSDFYKACMDRDHRNEIGATEFLNDLKKFGGWPLIEEEWDAANFNWSNVMIQ<br>LFKNGYISNMLFTVKLEIDAKNTSRSIIAVMQPSLGLKNKLYYIIPDGQIATSYKKAIFEVAKHLHPNANLVKKTSMDAIEKAFELESKIADLIKEVNMRYPDQFY<br>NIMTLEELENFAPKIPWRNLLKHLLPPETILTNEERILVTVPSMIKGLNKILEEMSTEQGKRILSNYMFYRTAFFAASNLNTEFYNLIKGIFNTDSVANNQLWEIC<br>VKDTMDLFPLPLTSSYVAKYLKNNTKELLEVMFDDIKNALVEDIKNAAWLDTETKNYALFKVKKMKAAIAYKEEIMKDENLNSFYKEVNVTDNHFSNVKQLIA<br>ARQQRNMQGLHKKRDRFRWTDVNNLLTANAYYQPSSNAIIIPVGVLHDTIFAAERPNYINYGSLGMIVGHELTHAFDSIGSLYDADGSYRMWWKKESWKK<br>FEERVKCYKEQYDKFYLSEADSNVNGTLTLGENIADNGGLQTAYYAYNLLLKRLGSEKSLPGLPYTERQMFWISFANVWCRKQTEESIQYGMIYSVHSPS<br>RFRIIGSLSNMKEFSEDFNCKLNSTMNPKSKCLLWK	Neprilysin-1 (W4VS99, *Trittame loki*)
m.60402	MHNNKGGDEEKTDPKENDKCMENNEHDKDSEVNQKSSKLDALRGRAKQLASNKYHLIIVLLCVLLFILFLIVIILAVQLGTYACEVPNECRTADCLRAASFVL<br>DRSEPSEDACDDFWAYSCKNWISQNPVPANKGSYSVTDELQDKIYNRIRHLIDLIRHDVDSSSIEMKVKTFYDSCRNMHNIERRFPDDIKRAIYDIGGWSLIE<br>YARPLQWDRMEVLSDLHSVYGIPVFFQVVVEPDDLDPQRNIIKLIPGGLGLPNRDYYFRPHDDTYVKAYKTFMIDTVKEMGVGLSKANQFADEVFNYEKRI<br>AEITPSTEELRSNPNLYVVKTIKDLDIPPSVIQWTKFLQAYFKDSADPETEIGVLSENYFLDISRIISSTADSVLNNYYMWRFLHTFVPFSSSRFRLVANYFKQT<br>FEGVPDLNLKYKENWESCIDETVKYLGHAVGAMYVSHYFPRESQDEIQLYLSKLAGAFVSVSNNMPWLEEEHSSALSKVKSLLLLSGHPAFIKNNTLNSYY<br>KELRVSIDFLKNIESGVHFLHKKQEEMLKSRATTDYSWTIYSHDVMAHYKYAGNQLVVPAGLFESPLFDSKSPLSVRYGVLAAHVANKMAEAFDDKGINYD<br>EFGAIKNWLNNDTHLSFASRKKCLKSVISNSFIDDIQANADLTIGSFIADIGGIKIAYEAYKRHAEEDQEFARLPNVGVSNKQAFFISYAQSLCQSIRPEKLKSY<br>RDANVELPAELRVLSTLQQLPEFSEAFSCSRDRSMNSQEHCRMW	Neprilysin-1 (W4VS99, *Trittame loki*)
m.28887	MAVTVTQSQLTLTSGRDRFRNPPWWNRRSRLERILCGVAGICLLMCTAMAVALAVVGYHYQVMKTGNGVNEAGLPSKTDKLVYSLYNAIYPPKEIGICLTP<br>GCVKAAATILNNINEKVEPCENFYEFACGGWLQKQLIPDDRSSVSVFSLLQDDLDQLLRSMVETGIKDTDPQYIQNLKSMYESCMNTTHIEMMGNEPLLRV<br>VKELGGWPAVEGDKWNSTNFDWMEALFALRKVGFGHNIFLSVSIGPDIRNNTRHIVDLDQASLGMPDRNYLLKGVEDPLVSAYYQLMVDSAVLLGANKTK<br>AQEEMKSALLFEIAIANFSVPREERRNISRLYNKMTVDDLYKLAPDLNWDKYFNKLLMDKISRSEEVIVIVPDYVQNLEKLMLSTDKRVIANYLMWRVVGQAF<br>PTLHRAWGEISQHYSSILTGKVRQEARWEHCLGTLSGSLSTALASLYVRHHFQDGSKDLALEMVNYIHREFLHVLSDVDWMDSQTKERAKEKAQNMATYI<br>GYPNELLEEWKVSEIYDGLHLNSSSYFENIRILRKWATDYVLSKLRKPNVKGDWKKRSAAAVVNAFYNSIENSIEFPAGILQGVFFSKDRPNYLNFGAIGFVI<br>GHEITHGFDDRGRQFDKDGNNINWWEPETDSTFRERAQCIIDQYGNYSVDEVGLQVNGINTQGENIADNGGIKEAFRAYLQWIRDHGAEDYLPGIKYSPT<br>QLFWISAANVWCGKYRPEVLKLRIMTGSHSPAPYRVIGPLANTPEFAAEFGCPLGSPMNPVKKCTVW	Neprilysin-1 (W4VS99, *Trittame loki*)

m.43613	MFIDDEMPRYKRTDFEEEETSSMASMAVDAPMNPPSSQGVQVRYSPGRRRNIWERASPLEKILVVCVLVLVLLVIILSAVLNSYSSSGTVKVVHITAKNGSSSGNNATEYCVTPACVTVAAAILNAMDQTVDPCTDFYQYSCGGWIRSNPLPDDKSIWGTFGKLWQENQIVMKNVLEDDSFKLTSEAERKARIYYMSCLDKNETVDKLGAKPIIDLLSKIGGWNISGDFNISQWNFQRTLELLHNQYNRGGLFSWGVGEDERNSSRNILQLDQGGLGLPTRDYYLNKSKDDEVLVAYLNYMTKVGVLLGGEENATRAQMEDVIEFETKLANITIPADERRDDEELYHKKTLNELRDLAPVLDWASYFNSAFRRINREITPAQELVVYAPEFIHKMSELVTQYLSSNEGKVVISNYLGWSLVQSLTSCLSKPFREASKILRKALVGSEGGESPWRYCVSDTNEVIGFALGAMFVREVFHGDSKPMAENMINEIRDAFKENLPLLKWMDPETRKLAKEKADAITDMIGFPEFILDPKQLDKKYEGMDFSEDEYFNNNIEVGQFALLSNMRKLDKPTNRSEWKMTPPTVNAYYTPTKNQIVFPAGILQAPFYDPNYPKSLNFGAMGVVMGHELTHAFDDQGREYDKYGNLHQWWKNSTIESFRERTQCIVDQYSSYEVNGESLNGKQVLGENIADNGGLKAAFHAYQDWVKSHPIELPLPAVPLSNNQLFFIGFAQVWCSTSTPEAMHLQILNDPHSPAKFRVVGTLSNSVDFAREFKCPPKSAMNPENKCEVW	Neprilysin-1 (W4VS99, *Trittame loki*)
m.252247	MYDYDIALLQMERPAEYNEYVRPICLPPKELPDDSPLYKPKSSAWATGWGHDAKISRNDRVIPRTTENLKELYLPIQSKERCIESTRSNGVNISFFTDRMFCAGDGSGGNDTCQGDSGGPLMQAQTNAEGYIYWTQVGIVSWGIQCGMPNTYGYYTYLQRFVPWVEETIENALGAVGM	Venom peptide isomerase (Q9TXD8, *Agelenopsis aperta*)
m.77294	MIFHVSLVTAISLLFGIKYPVFMTHGVFNISHDLALARLNAPVLMNPHVQVVCLPQLNEELQAGERCFATGWGATRGSKSENVLKQAEHPIQDETKCYNEYQKFESETMICSGRLEPLHGLCHGDSGGPLVCKKDGKWHVYGVASFITDTNFISGLCGLANKPTVFNKISAKVEWIKSIISNNN	Venom peptide isomerase (Q9TXD8, *Agelenopsis aperta*)
m.183905	MSSGEIVCSGTIINKRFVMTSAACVTTSKSIPKKPNEISVVLGLSNRNGGDQSQRFGVKAIYVHPGYRTLNYDNDIALLELEKPMVYSRYIRPICLPTDSSNLYKPQSPGLGLGWGSTSPWGNAGTLKRNSLKEIKVTIQHPDACKEYLGEKYARYFTNLVFCAAEESPALCSGDSGGPVMQSEMREDGHRIWRQVGIVSFGMGCGPNNSMGFYTYVVYKFLPWIQDIADNSN	Venom peptide isomerase (Q9TXD8, *Agelenopsis aperta*)
m.183910	MAAVFYVKDSVTKDDIVCSGTIINKRFVMTSAACVTTSKSIPKKPNEISVVLGLSNRNGGDQSQRFGVKAIYVHPGYRTLNYDNDIALLELEKPMVYSRYIRPICLPTDSSNLYKPQSPGLGLGWGSTSPWGNAGTLKRNSLKEIKVTIQHPDACKEYLGEKYARYFTNLVFCAAEESPALCSGDSGGPVMQSEMREDGHRIWRQVGIVSFGMGCGPNNSMGFYTYVVYKFLPWIQDIADNSN	Venom peptide isomerase (Q9TXD8, *Agelenopsis aperta*)
m.61171	MFTAICILWSLQLIQGSPLDLSLEDTRPLCPCGDYASSVNTRVVGGHAVPKHEFPYASNLLFRPFEEYSSRQASQKLSPFCGGTLITDRHILTAAHCLKDRDPEDLAVDVGDYSLRELKDRQVVNVRNVTKFPEYRKDSFHTDIGIIELEHPVRFRQGMRTALLPNEGLKLKPGTTVSVYGWGRLSYYGGHPDVLNTVDLPVVRNEECQHEFISRIEPSMICAGGQEGKDACIGDSGSGLIVRLDNEFVLCGVVSFGRKCALPHVPGVYTRVSSYTDWILDQTRSASCRPCVYDE	Venom peptide isomerase (Q9TXD8, *Agelenopsis aperta*)
m.71477	MFADSKRPRFCMLHRNTTLNSYFLLSILFTLGCVAKGGRPKTVKNCGIVDVPQARIIGGIWAQHGDYPWMVSVHEYYRGFDHVCGGTIINEHWILTAAHCIDYPTKPRKYEIYVGLHRLSKKDRKVSKKHKISKVIVHEEYDKETFLNDIALLRTKEPINFRASRGYVNGICLPVTDKDPKGWAIVTGWGHTLEGGDNSDVLKEVKVPIVPRELCNEAYDDDPDDDIEENIFDTQLCAGAPNRDSCQNDSGGPLIQRSKNGVHTLIGVVSYGSGCGDRHFPGVYTKVASYMDWMYKNMKD	Venom peptide isomerase (Q9TXD8, *Agelenopsis aperta*)
m.4971	MQLVFAAFLLLLTGYTSGKLSTVKDCGKATVPQARIVDGEVSKRGKYPWMVSVQQWDGDKLRPICGGAILNENWIVTAAHCFDQPVKNSDYEVYVGLFSITKTNEPPVRKHKVSKIIIHEDYKDTGFANDIALIKTATPIDIKGSKGYVNAICMPSGITNPAGDAIVIGWGTIYDDGPLSAELREAVVPIVPWKKCKEIYGNINSDFEYVQVTPFVMCAGGTGKDSCQGDSGGPLFQFDKNGVATLLGTVANGGDCGNGRYPGMYMKASAYKSWMDRVTT	Putative PQM protease precursor (AUI10826.1, *Geolycosa vultuosa*)
m.4960	MQLVFAAFLLLLTGYTSGKLSTVKDCGKATVPQARIVDGEVSKRGKYPWMVSVQQWDGDKLRPICGGAILNENWIVTAAHCFDQPVKNSDYEVYVGLFSITKTNEPPVRKHKVSKIIIHEDYKDTGFANDIALIKTATPIDIKGSKGYVNAICMPSGITNPAGDAIVIGWGKIYDGGPISAELREAVVPIVPWKKCKEIYGNINSDFEYVQVTPFVMCAGGNGKDSCQYDSGGPLFQFDKNGVATLLGTVANGGDCGNGRYPGMYMKASAYKSWMDRVTT	Putative PQM protease precursor (AUI10826.1, *Geolycosa vultuosa*)

(7) Metalloprotease

Name	Sequence	Similar protein (Accession No., species)
m.136344	MKVINWNIFVMNLLAEDPMVTEGFFEGDIDTENSFQMLNGISDDNLRWPNAIVYYAIEENLHHIKPLIQKAMQHIESKTCIGFVERTNEADYVWIHIGKGCSA<br>QWGRRGGKQRLSLGHTGCHTLGVVVHELMHAIGFQHEHSRSDRDDYITIFLENVRPGQEHNFEKLEPSENRLLTQFDTSSILLYGSTTFARPNTYSMLLKD<br>GSVMVEVYKKNSMTNLDAEKVNKLYQCT	Astacin-like metalloprotease toxin (A0FKN6, *Loxosceles intermedia*)
m.44881	MVAALVFILLSIGCSLVTSKAYLGDLPIQNPDLFGGDILGIEDAEDRNAIVDKRQIWPGGVVPYEQDPGLKATVFRLTLEGAFDQYKKSTCIKFVPRTNEKDYI<br>RLFPGEGCYSHVGRTGGQQPVSLGQGCGWMGTIVHELGHAIGFYHEQNRSDRDDWLIIYWENVKEGMEDQFFKLKPDQNQLLTPFDYDSIMLYGSYTFS<br>KDRKKLKTMVGKNNEFLQEVISKYRLSKSDIQRINTLYNCKM	Astacin-like metalloprotease toxin (A0FKN6, *Loxosceles intermedia*)
m.35058	MAPFFYFTCVVLWLGSSSATEDFLGDLPMQNPDLFQGDILGVEDPEDRNAIVDKRQIWPGGIVPYVLDPGITKTVSPAILTNAMWLYKRDTCIRFVPRTNEA<br>NYIRIFPGQGCYSHVGLTHVGAQPVSLGQGCGYMGTVIHELAHALGFYHEQNRSDRDDWLTIYWDNIKEGMADQFMKLKPDQNQLLTPFDYDSIMLYGS<br>YTFSKEYGKLKTMEGKGGMFLKDVIRKYFMSKSDIQRIKALYKCPS	Astacin-like metalloprotease toxin (A0FKN6, *Loxosceles intermedia*)
m.220610	MFALAALSLFLVITTGLSRPEKDGPNVHPEYLDHLLDTEQYELFAKVMNIPDASLVRPKEGERSENPMFNKDLEMGDIVPAFGPLKEESRQGINFDRYPGS<br>KWQNGELHFYIKLNDFTESQLQTIYDAIDSWNRVVRCLRLIDLRETVRKISDYVYVFGGNGCWSYLGRIGGEQGLSLQPNGCVWKGTVLHEFNHAAGFAH<br>EQNRLDRDDFIEMLWGNIPDDWKSQYEKTNPRDYGLQCSYDYYSIMHYGIKAPGTNKDAFRVKQDGIDQNKIGRSTEFTKIDIEKINKLYC	Astacin-like metalloprotease toxin (A0FKN6, *Loxosceles intermedia*)

(8) Acetylcholinesterase

Name	Sequence	Similar protein (Accession No., species)
m.101805	MRRDTLLFCLLYLSYLLPCRSLLSRRVVTTPSGRVEGTVQSHNGVSVNAFLGIPYARPPVGDLRFKAPRTPAPWEGTLSAKEMRSGCVQFTRNPFPWRD<br>VPPGLSEDCLYLNVWAPSTLLNTINRRTVMFWVYGGAFLFGSSRVDFYDGHVLAVEGDVVVVTVNYRLGPFGFLYAGNEDAPGNMGLLDLVEALKWVRG<br>NIASFGGDPNSITLFGQSAGGIAIGMFCVSPLTRGLFKRVIMQSGSPTNLGGEDNNLDAKMSQRLAEAVGCAEDENSLADDPETVVECLRNVDAFELARTF<br>SALGGNGSRLVYPRFGDELLPQNPRKAIAKGDFGAVDVLIGSNSGDGALIITDSMTEVFGFFGEKDPQINRTFGTSIIKRQFQDFVAPCSVVKQYLGGVQDD<br>DFDGIRQQVFMATGDYERTCPATYFAESFAKKGVKAYYYFFTHRPSKSPFAPWMLASHFDETPFVFGYPLRYTRNYTIRERLLSRRMIQAWTAFAKTGTPI<br>GGVWPQYSRENPRYQILDVNSLGTGMGPRQNNCDFFRPFFGF	Acetylcholinesterase-1 (W4VSJ0, *Trittame loki*)
m.126349	MRLFLLLSIITLASCDRTVTTTNGPVKGISLSPKKVVAFLGIPYAEPPVGPLRFAKPIPKSFSEEVYLANEFPPSCVQASLGDFYFAPDPKGKMSEDCLYLNV<br>WVPEGGSDTKPIMLFIHGGAFMVGSSNMKVYDGANLSEHGDVIVAAINYRLGTLGFFSGFVEDAAGNMAMYDQVMAIQWIKNNAKHFGGDPDNIVLFGQ<br>SAGGFSTSLHLISPLSKGLFKRAILQSGTVVSPLFKSNDVLQMTSQYLASFLGCTDENNSLNKNPQSVIDCMKALPTEKIIQADGILLKSGGMMFPRIGDSFL<br>PENSVDSYKNGDFKDTEILLGVTSNEGSLFLVFHMTDTLGSFGQKIEVQDFNETSAMQYFKRISQHNSKGMPSEIVQEYFDRVKKDPGYTYLQATSDLMG<br>DIIITCGAVFQADYYSLRDNPVYFYVFDYRPDSTPLAEWMGVAHFDEMQYVFGNPLHQEFTEYEKEFSGDIMDMWVAFAKTGNPNIPGGVKWPRYSYQD<br>PKYLVLSKRDRVAVKPDNNSCEKWRKVFETEIKEDTLKKLKKPA	Acetylcholinesterase-1 (W4VSJ0, *Trittame loki*)
m.229987	MRLVCLLLLFGCTLAFDYEGYGCNDNPYIRDAGPLVRTSSGSLFGKYVKVFDTKVAAFLGIPYASPPVSEKRFLKPTPLERWDGVLDATVKPPPCMQYSSR<br>NFSWIPKSTPSEDCLYLNVWTPAKCCCTGSERLPVMVWIYGGGFYSGSTDMDVYDGSTMVSYSKAIVVSINYRVGIFGFLSSGSDEAPGNVGLLDQNLAL<br>KWIKKNIGYFGGDPERITIMGESAGAMSVSLHMVSPMSRNLFQAAILQSATAYQPQMVDNKAMSYIKANTFAKVIGCLQGNSSIESDPEVMECLKAKSSWE<br>LARAEDVFTSKLPQYFLPTYGDEFLPTSPFKAFNEGHLAPVDILIGNNRDEVTIFLNYMVPDIFRLDSEPQLNLEAAKQIIHGLFQTVPRPSATEIFDHYFQNV<br>SEGDSSEIVKTIAHAFGDYVFYCPVALFAEKARNAYVYWVSHRSIRDQNPEWLGVPHFYEVPFVFGVPIQDTFTYTPEDGRFSADLIKKWTTFAEHGHPLG<br>DRNPDDWPPYSAENPMAYELNPRQRGLLSFPRADACEFWRPYIVPKTC	Acetylcholinesterase-1 (W4VSJ0, *Trittame loki*)
m.130212	MKFLFIIQSCFTVVTFRLLFAPVFGEESNFGQQNSRYFAEPEFKYYTTQNFRYSYTEFDHAAHDERCGPIYESKPTHKITPLGIVRGRQVFLCDRPGIPLRQR<br>PGQIGKYIHSVQVFLGIPYAEPPVRRRNEENLQFKEPLEKQKFIDIDASSYKPACPQHKKYFSKGQGINTTSEDCLYLNIFSPVQEDSDSLLLNGYPVMIYIHD<br>GNWDHGSGALFPGHMLAASQKVVVVTFNYRLGPLGFLATGDSNSPGNYGMLDQVAAINWVYQYIKAFDGDPDKITVFGSGVGGASAALLALSRLTKRKIK<br>RVIAQNGSPVADWAAISDPRFMQNTTEIVGEGLGCSQQDTAHLVECVGTRLNEIKILEIKPRIGWLPWGPVVDNFTRRSLDQFLHESPEDMLEKGIDFRKD<br>FAFMTGVNRDQGASIFLEDVITYRDLDITSQVFDKKIRSFMKMYNYSVNAEGIFDAIKFMYKPPLDSYNETLLREAYIHLLTDRYYVAPMEKIVHLMVKNKVPT<br>YAYVLNYTLQGYSTILKDFISPEVDYLLLSGAPFMDPKYYPAYLNLKNAKWTEADRNMSQAMMEAWANFARDGEPTKTRLFNTIMWEKVEENYLRYLNFN<br>ATNTSSEMIYKYRERESRFWNFFLPFFIDREPPTLAPTLEPGVAELRLLTSALWGSVFVAVLLIVITLVCCSLYCRVRRYDSY	Acetylcholinesterase-1 (W4VSJ0, *Trittame loki*)
m.130210	MKFLFIIQSCFTVVTFRLLFAPVFGEESNFGQQNSRYFAEPEFKYYTTQNFRYSYTEFDHAAHDERCGPIYESKPTHKITPLGIVRGRQVFLCDRPGIPLRQR<br>PGQIGKYIHSVQVFLGIPYAEPPVRRRNEENLQFKEPLEKQKFIDIDASSYKPACPQHKKYFSKGQGINTTSEDCLYLNIFSPVQEDSDSLLLNGYPVMIYIHD<br>GNWDHGSGALFPGHMLAASQKVVVVTFNYRLGPLGFLATGDSNSPGNYGMLDQVAAINWVYQYIKAFDGDPDKITVFGSGVGGASAALLALSRLTKRKIK<br>RVIAQNGSPVADWAAISDPRFMQNTTEIVGEGLGCSQQDTAHLVECVGTRLNEIKILEIKPRIGWLPWGPVVDNFTRRSLDQFLHESPEDMLEKGIDFRKD<br>FAFMTGVNRDQGASIFLEDVITYRDLDITSQVFDKKIRSFMKMYNYSVNAEGIFDAIKFMYKPPLDSYNETLLREAYIHLLTDRYYVAPMEKIVHLMVKNKVPT<br>YAYVLNYTLQGYSTILKDFISPEVDYLLLSGAPFMDPKYYPAYLNLKNAKWTEADRNMSQAMMEAWANFARDGEPTKTRLFNTIMWEKVEENYLRYLNFN<br>ATNTSSEMIYKYRERESRFWNFFLPFFIDREPPTLAPTLEPGVAELRLLTSALWGSVFVAVLLIVITLVCCSLYCRVRSYKRPPDDLDSSREVIVNHSATQQD<br>TAV	Acetylcholinesterase-1 (W4VSJ0, *Trittame loki*)

(9) Cysteine-rich secretory protein

Name	Sequence	Similar protein (Accession No., species)
m.230427	MHIFSLFLSITILVTYAVAQTNTECPELFLRYSSTHSYCKTDNPKCNILRSFINEEEKNTIVRVHNSYRSRLAIGEEQNLPTAGDMLQMVWDDELAAVAQKHA<br>QQCLFEHDCANCRRVQNFGVGQNLAIEQVFNSPSVPNSNWPSAIKSWYDEIAYFSSNAIDPFIAPPPEPTYGHFSQMFWASSWRIGCGYVLYQDRNMYQ<br>KLYVCNYGPAGNTVSQSMYKVGSPCSACPLNSCCDKTCGAKAEYSGLCKITDNKAPKYKPVVPYIFYCDFNNQSDCQSYVTGVNKWLVYNTLSGSFLSIIL<br>KGGEESSMTFQNKIKPSTSAFCFVVMYRKGPNEDGKEEASTATETFSGSGFEFQQNLPTFNGDVKQQFSQFSMTLSWNMETEIKITFAVPAGKPDQFFEI<br>QSIYATEGLCKQ	CRISP/Allergen/PR-1 (W4VS53, *Trittame loki*)
m.45429	MSAPLGIPSMFLALCALLCVLVAVQSAYCPDPYKRYSKDHSYCVGVCCSCAKYRRGVSPWLKQELVRLHNNLRSKVAGGKSYGVDHLPRATNMLEMVW<br>DDELAEVAQRWSDKCERKSDCEDCRRVGRFGVGQIIFDFDGKMEGEDIENEFFSRFQGLQSFRKEQVERFTAGTNPKTAQMLWAKTWRIGCGFLDFNY<br>PGNGRRYTQIVCNYGPNGNVEGEEVYKGGSVCSACPANTCCGDSCKKYNLKANYHGLCKVIDENLPPEGNVPHKKTGNEVFYCGFNDESDCSHSVEGV<br>DRWIRNVTTGGTWLNTYLGNYGHTVLKFNQPIISKSGKLCLAIKTRSGPMEAGQPYRYQLSGGLEEEGKFSTSFVFPPADDNVKHKFHTDYFQPSEFPKN<br>RGAKLTLSFQVSGEGPKTYLEIDSIVALEKDCPKKTITEGTGFITSP	Venom allergen 5 (A9QQ26, *Lycosa singoriensis*)

(10) Techylectin-like protein

Name	Sequence	Similar protein (Accession No., species)
m.72849	MSFSSNISDEAQASIVVTLLNNARNLLDDAKKYATMKTKLSAAENVYYRPMDCAEVREKNPLARSGVHKIWPHSRAVNGSVEVYCDMEDDGGSWTVIQR<br>RGDFDSESDYFFKEWQFYKVGFGDRMKDFWLGNDVIFALTNQRLN	Techylectin-like protein (P85031, *Phoneutria nigriventer*)
m.257368	MIFALTNQRLNEVKFTLTDWESNTTYATYDEFWIDDEDHKYTAHVSGYKGTAGDSFYNTNGMPFTTKDQDNDKYEKNCAVQSRGAWWYGRCHMSNLN<br>GMYLKGFHESNADGVNWYSLRGHHYSFKDTVIKIRPKGFQKNQCGTTSL	Techylectin-like protein (P85031, *Phoneutria nigriventer*)
m.72846	MLSRLLVWVNLMGVMYLGIYADDPQFISPLSRNITDQTQSSIVVTLLNNARNLLEDAKKYASIKTRCYAAENVYGRPMDCAEVKEKNPLANSGVHKIWLHS<br>RAVNDAVEVYCDMEDDGGSWTVIQRRGDFDSGKDYFYKDWQFYKKGFGDRMKDFWLGNDVIFALTNQRLN	Techylectin-like protein (P85031, *Phoneutria nigriventer*)
m.72851	MLSRLLVWVNLMGVMYLGIYADDPQFISPLSRNITDQTQSSIVVTLLNNARNLLEDAKKYASIKTRCYAAENVYGRPMDCAEVKEKNPLANSGVHKIWLHS<br>RAVNDAVEVYCDMEDDGGSWTVIQRRGDFDSGKDYFYKDWQFYKKGFGDRMKDFWLGDSFSAVNGLPFTTKDQDNDKYEKNCAVQYTGAWWYGSC<br>HLSNLNGKYLKRVHKSHADSVNWHGFRGHNYSLKDTVIKIRPKDFQKNLSGITPL	Techylectin-like protein (P85031, *Phoneutria nigriventer*)
m.265263	MINHWVPVMGILLFCTDCGAADSTCGEKVSLYYHDAAADLIAKAKYHFPSRLHCENLSRNAVDCSEWLEKGYKKNGVYTVWPQSRVVDGKPLEVYCDIET<br>DGGGWTLIQRRGSFGRAVDYFYKDWKSYKEGFGDTEKEFWLGNDNIFALTNQGLYSARFDMKDIDNETRYAHYDKFWIDDEYNKYTLHIADYRGDAGDS<br>LVEHHNHQKFSAKGEDNDNQKDENCVEKYRGGWWYNSCHESNLHGLNLKGKHESFANGINWKSWKGYYDSLQSTEIKIRPRNFKLPHSSLNNTPM	Techylectin-like protein (P85031, *Phoneutria nigriventer*)
